# Rapid runtime learning by curating small datasets of high-quality items obtained from memory

**DOI:** 10.1371/journal.pcbi.1011445

**Published:** 2023-10-04

**Authors:** Joseph Scott German, Guofeng Cui, Chenliang Xu, Robert A. Jacobs

**Affiliations:** 1 Institute for Psychology and Centre for Cognitive Science, Technical University of Darmstadt, Darmstadt, Germany; 2 Department of Computer Science, Rutgers University, Piscataway, New Jersey, United States of America; 3 Department of Computer Science, University of Rochester, Rochester, New York, United States of America; 4 Department of Brain and Cognitive Sciences, University of Rochester, Rochester, New York, United States of America; University of Tokyo: Tokyo Daigaku, JAPAN

## Abstract

We propose the “runtime learning” hypothesis which states that people quickly learn to perform unfamiliar tasks as the tasks arise by using task-relevant instances of concepts stored in memory during mental training. To make learning rapid, the hypothesis claims that only a few class instances are used, but these instances are especially valuable for training. The paper motivates the hypothesis by describing related ideas from the cognitive science and machine learning literatures. Using computer simulation, we show that deep neural networks (DNNs) can learn effectively from small, curated training sets, and that valuable training items tend to lie toward the centers of data item clusters in an abstract feature space. In a series of three behavioral experiments, we show that people can also learn effectively from small, curated training sets. Critically, we find that participant reaction times and fitted drift rates are best accounted for by the confidences of DNNs trained on small datasets of highly valuable items. We conclude that the runtime learning hypothesis is a novel conjecture about the relationship between learning and memory with the potential for explaining a wide variety of cognitive phenomena.

## Author summary

Human cognition is remarkably flexible, with the ability to reliably perform a wide variety of tasks, including idiosyncratic and unfamiliar ones, in a wide variety of contexts. However, despite impressive advances in machine learning, leading to artificial intelligences capable of outperforming humans on select individual tasks, this flexibility has yet to be replicated by computational models. Doing so is essential to both creating general artificial intelligence and to achieving a deeper understanding of the human brain and mind. To account for this vital property of human cognition, we introduce the “runtime learning hypothesis”, in which the brain rapidly constructs task-specific models in response to the needs of the moment using an internal training process based on valuable exemplars of the relevant concepts. We examine the plausibility of this hypothesis using human behavioral experiments and machine-learning-based computational modeling. The results are consistent with the human brain using runtime learning to flexibly perform tasks.

## 1 Introduction

Perhaps the most impressive aspect of the human brain is its flexibility—it is able to handle a multitude of different tasks in a multitude of different contexts. In fact, it can handle so many tasks that it is implausible it has dedicated neural networks for each of them, especially considering it can even handle tasks it has never encountered before. There are simply too many obscure tasks people can complete with ease. Are we to believe that brains have networks dedicated to determining if an animal is safe to pet, identifying the creator of a painting from its style, and deciding which book at the library seems most interesting? Do people have networks dedicated to answering questions like “During what decade was this photograph taken?” and “Which of these cars is the fastest?”. Yet people are able to give sensible answers to these and many other questions. If human brains do not have neural networks dedicated to these tasks, how is this possible?

Even for tasks that seem common and important enough for a dedicated network, the situation is more complicated than it appears. Consider the task of determining if a scene is from a natural environment, such as a forest, or an artificial one, such as a city street. Such scenes vary significantly in appearance depending on numerous factors such as viewpoint, lighting conditions, and the medium and style of depiction (e.g., a photograph versus an Impressionist painting). People are faced with either the daunting task of acquiring a single network that is invariant over the multitude of combinations of these factors, or of acquiring and storing many networks so that hopefully one of them will be suitable for the specific task at hand. The former is extraordinarily challenging, as decades of cognitive science and artificial intelligence (AI) research have shown. The latter seems monstrously inefficient, as it uses precious resources to prepare for tasks whose narrow scope means they will rarely come up.

In essence, then, it seems that both people and the artificial agents studied in the field of machine learning (ML) are faced with the same problem, namely how to quickly learn to perform novel tasks in the absence of task-specific training information—also known as the problem of “zero shot” learning. The key hypothesis of this paper is that people are able to rapidly learn to perform unfamiliar or idiosyncratic tasks at “runtime” (i.e., in an on-the-fly fashion as novel tasks arise) by internally training neural networks using carefully curated small sets of high-quality data items obtained from memory. We elaborate on this hypothesis in the next section.

### 1.1 Runtime learning

Instead of attempting to produce extraordinarily robust networks that are invariant to contextual variations, we propose an alternative: when faced with tasks for which they do not currently have dedicated neural networks, people quickly generate new networks then and there, a process referred to here as “runtime learning”. How might people quickly generate these new networks? A clue to the answer to this question may be found in exemplar theory, which proposes that people recognize and reason about concepts and categories using exemplars (i.e., previously experienced stimuli) stored in episodic memory [1–3]. Although these exemplars can be used to train neural networks, training a network using all exemplars stored in a person’s episodic memory would be extremely inefficient, as this memory may contain an enormous number of exemplars, most of which are irrelevant to the task that currently needs to be performed. Consequently, we conjecture that people are able to identify a (hopefully small) subset of their stored exemplars that are task-relevant, and then use this subset for rapid network training. For instance, if a person is asked whether an image is of a fox or a wolf, the person can marshal the best stored exemplars of foxes and wolves and use these to train a network that answers the original question.

In essence, we are saying that people’s brains treat many or even most tasks as zero-shot learning problems, which they solve using an exemplar approach in which a neural network is trained using exemplars drawn from memory. “Drawing from memory” may take various forms: the exemplars may be representations of actual exemplars that have previously been encountered, or they may be entirely novel exemplars synthesized by a generative model of a concept, or some combination of natural and synthetic exemplars. We mostly leave the question of exactly how exemplars are selected for training for future work (though see below for ML research on this topic).

Although the exemplars used during this internal training must be based on prior experience, this does not mean that the tasks they can be used to solve must be familiar. Novel tasks may use unfamiliar groups of familiar categories. For example, although one may have previously encountered rabbits, tractors, and unicorns, specifically needing to discriminate between these categories is probably an unfamiliar task. This is not a trivial generalization—the features needed to distinguish rabbits, tractors, and unicorns are surely not the same as those for distinguishing rabbits, squirrels, and mice, or tractors, cars, and trucks, or unicorns, hippogryphs, and centaurs.

If an agent has a knowledge base that includes hierarchical and relational properties between concepts—as found in semantic networks, frames, or graph neural networks, for instance—the system can address tasks involving unfamiliar categories that can be constructed from familiar ones. For example, if one is familiar with rabbits, foxes, squirrels, etc., and knows they are all mammals, one can construct a “mammal” training set from these subcategories (e.g., by using symbolic or graph neural network data structures and reasoning operations of the type that have been studied in cognitive science and AI for many years [4–8]) without needing to store instances of a separate, distinct mammal category. Acquiring knowledge such as “foxes are mammals” is relatively easy (e.g., one need only read it in a book). It should also be possible to reorganize the knowledge base to account for new information. If, for example, we have stored instances of cats under the generic name “cats”, and if we learn that “Siamese cats have blue eyes and point coloration”, then we can check our stored cat instances, find the ones matching the Siamese criteria, and segregate them into a subcategory in case we ever need to perform a task involving specifically Siamese cats.

The prospect of learning to perform an unfamiliar task only at the point the task is presented may seem implausible due to time limitations. However, we are inspired by recent cognitive science and ML research on rapid learning. In the following section, we describe this research, along with other relevant background materials.

### 1.2 Background and related work

Efficient, rapid learning has been studied in both cognitive science and ML literatures. For example, methods to achieve rapid learning studied in cognitive science include rote learning, imitation learning, and active learning, whereas methods to achieve rapid learning studied in ML include dataset distillation, meta-learning, and few-shot learning. We emphasize that these methods are typically not mutually exclusive. That is, an agent—biological or artificial—could simultaneously use multiple methods to achieve rapid learning.

One of the focuses of the runtime-learning hypothesis is achieving rapid learning by curating small training sets of high-quality items obtained from memory (as just emphasized, people undoubtedly use other methods too). This focus stems in part from the belief that this is a potentially important cognitive strategy for which there is relevant research in the ML literature, but the hypothesis is not currently part of cognitive science. In this section, we briefly review some of the research most relevant to the hypothesis in the ML and cognitive science literatures.

#### 1.2.1 Machine learning

Some aspects of runtime learning are similar to, or can be combined with, techniques from the machine learning literature. We briefly review some of these techniques; interested readers can consult S1 Appendix for further details, along with other relevant methods.

A core tenet of runtime learning is that models are trained on only a few exemplars. ML researchers have created methods of identifying or synthesizing category exemplars that are especially valuable for training algorithms, usually by treating the training data as a parameter to be optimized, in a process variously referred to as “dataset distillation” or “core set selection” [9–14]. The resulting training set is expected to produce models that perform much better than would be expected given the amount of training data, sometimes even comparably to models trained on larger training sets. It is also possible to train generative models to produce particularly valuable synthetic exemplars. (Generative models learn the joint distribution of classes and observations, and when combined with a prior distribution over classes, sampling from a generative model of a class produces samples that are likely to belong to the class. See S1 Appendix for more details.) [15] created an algorithm based on this concept called “generative teaching networks”, in which a generative model is “meta-trained” to produce exemplars that can be used to train a neural network to a high level of performance.

Separately but complementarily, performance when trained on only a few exemplars can be further improved with “meta-learning” techniques. In meta-learning, a model “learns to learn” so that it can be trained on novel tasks more quickly, reliably, accurately, or otherwise “better” than it normally could [16, 17]. Approaches include learning special learning rules, learning to better characterize tasks, and learning features that transfer to other tasks. Like dataset distillation, meta-learning aims to make learning efficient, but instead of operating on the training data, it identifies effective initial conditions.

#### 1.2.2 Exemplar models of concepts and category learning

Many popular models of human object perception, recognition, and categorization are “exemplar models” [1, 2]. A representative and influential early model of this sort is the Generalized Context Model [3], which performs classification tasks by comparing test items to stored exemplars of the classes. Test items are assigned to classes based on their similarity—or distance in a psychological space—to class exemplars, where the embeddings of items in the psychological space are possibly learned and altered by attention, usually by modifying the weight or importance placed on particular features. Thus, exemplar models can judge the similarity of exemplars contextually by attending to the features that are the most relevant to current goals. Exemplar models are often contrasted with “prototype models”, in which knowledge of a category is represented by a single “prototype” formed by aggregating multiple exemplars of the category.

More elaborate exemplar-type models introduce a greater level of abstraction in their exemplar representations, effectively occupying a middle ground between exemplar and prototype models. Often, this amounts to learning multiple prototypes for a category, corresponding to the category’s clusters in psychological space. The Varying Abstraction Model [18] proposes that depending on the category structure, particular exemplars may either merge or remain separate, depending on the circumstances. The SUSTAIN model [19] can learn categories from sequentially presented exemplars, and adds new exemplars to its memory when it is unable to adequately account for them. It also has the ability to modify its current exemplars such that they better represent the concept. Thus, the SUSTAIN model learns both how many prototypes it needs, and what those prototypes are.

To extend these results to more complicated, naturalistic stimuli, exemplar models have also incorporated advances in machine learning, using deep neural networks to embed the exemplars into a psychological space, instead of the hard-coded spaces used in previous work. [20] found that cognitive models using feature representations from popular off-the-shelf DNNs outperformed those using hard-coded features on naturalistic stimuli. [21] trained their own DNN from scratch with ground-truth embeddings derived from another, more expensive method, and successfully used it in combination with the generalized context model to predict human responses. It is even possible to learn the embedding DNN and the exemplar model together in an end-to-end manner [22].

While runtime learning also makes use of class exemplars, it obviously differs in that it identifies and retrieves the exemplars at test time. It can also use the exemplars indirectly, as part of the process of learning to perform a particular task when the task presents itself, whereas exemplar models can only use the instances more directly as part of a “similarity” calculation, and exemplar models assume that the learned aspects of the decision process (such as a custom psychological space, or an attention mechanism that creates that space) exist before test time. This means that the decision process learned at runtime can be for an unfamiliar, possibly unanticipated task. Runtime learning thus postulates a much more extreme form of abstraction from the exemplars than even prototype models. Not only can the relative importance of features be modified for a given task, the features themselves can if necessary be learned, and need not be explicitly present in the exemplars drawn from memory or in the psychological space.

#### 1.2.3 Complementary learning systems and memory consolidation

The runtime-learning hypothesis can be compared to the concept of “memory consolidation”, in which short-term memories are converted into a stable, long-lasting form [23]. An influential theory of memory consolidation is the“complementary learning systems” (CLS) approach [24, 25] which, as its name suggests, proposes that the brain maintains two separate but interacting learning systems: a fast system that learns primarily by storing—or rote learning—instances or examples pertinent to the task being learned, and a slow system that consolidates this knowledge into a more abstract and longer-lasting understanding of a concept or task, in part by accessing the instructive instances stored by the fast system. In the brain, the role of the fast learner is often assigned to the hippocampus and associated structures, while that of the slow learner is fulfilled by neocortex. Recent research has suggested that the hippocampus is itself also capable of some degree of abstraction [25–27], perhaps akin to the multiple cluster prototypes in some exemplar models.

Electrophysiological recordings in behaving and resting animals have provided evidence for the basic concepts behind complementary learning systems. Patterns of neural activity seen in the place-selective cells of the hippocampus of behaving animals have been observed to repeat in sped-up form as part of sharp waves and ripples (SWRs) during rest and sleep [27–29]. Furthermore, these SWRs are synchronized with activity levels in the neocortex, which suggests that the neorcortex is integrating the information in this “replay” [30–32]. Interestingly, novel patterns of SWR of the same type as seen during memory consolidation, but not corresponding to any previous experience, are later observed during behavior [33, 34]. The function of these “preplays” is not fully understood, but one proposal is that they correspond to planning. Without necessarily contradicting this, we suggest that these may be indicative of runtime learning: the initial appearance of a pattern is the result of an endogenous training process, and the later reappearance of the pattern during behavior corresponds to the processing of an experience similar to those used for preparation.

Preplay aside, in the cognitive science and ML literatures, the complementary learning systems approach is usually conceived of as a way to improve the ability to learn from experience. Intuitively, this makes sense—maintaining a store of instances in the fast system allows the slow system to learn about both individual instances and relations among instances. Indeed, machine learning approaches inspired by the CLS approach have yielded impressive results, such as in deep Q-learning [35], which successfully learned to play several Atari 2600 games by using a replay system similar to the proposed hippocampal system. A CLS-inspired replay system is also used in some approaches to “continual learning” or “lifelong learning”, in which a DNN learns different tasks at different times, and must prevent training on later tasks from causing “catastrophic forgetting” of the earlier tasks [36]. A generative model of previously-seen concepts can be used to produce training exemplars that maintain performance on a previous-learned task [37–39].

Our hypothesis, while not at all incompatible with the complementary learning systems approach, can be distinguished in two important ways. First, while it also depends in part on learning from an internal store of instances, it functions primarily to learn tasks with which the learner does not necessarily have any explicit experience with whatsoever, instead of merely supplementing actual experience. For example, while a person may have learned the concepts of “cat” and “dog”, this does not mean that they actually have the ability to distinguish sensory input corresponding to these two classes, especially if they have no experience doing so. Our hypothesis describes how this “last mile” is achieved without explicit external training. In this sense, runtime learning can be seen as a “deconsolidation” process, which converts knowledge from an abstract, general form into one that can be used for particular tasks.

Second, while the “tutoring” of the slow system by the fast system in the complementary learning approach is usually considered to be relatively slow, and to frequently take place during the brain’s “down time” during rest and sleep (dreaming is frequently cited as a possible example of complementary learning in action [40, 41]), our hypothesis is intended to describe the brain’s fast response to an immediate and possibly suddenly relevant task. These differences suggest a way of combining runtime learning and complementary learning systems to form a learning system trinity: a fast instance-based learner, as in conventional complementary learning systems, as well as a generative (or otherwise abstract) learner taught, at least in part, by the fast learner, that learns to produce valuable instances, and finally a runtime learner that uses generated instances to quickly learn to perform the current task.

#### 1.2.4 Attention and working memory

An important implication of runtime learning is that preparatory adaptation is also one of the components of *attention*: one way of attending to something is by being prepared to process it. Indeed, under some circumstances one can interpret runtime learning as an attentional process, determining what task to focus on by the selection of training exemplars and initializations (which in turn determines the action of attention at lower levels, such as which objects and features are represented). This could explain certain types of inattentional blindness, in which unexpected, irrelevant stimuli are missed, yet are obvious when explicitly pointed out [42–44]. For example, in the famous “invisible gorilla” experiment, participants instructed to count the number of times a group of people passed a ball amongst themselves missed a gorilla walking through the scene [42]. Similar effects have been found in other experiments [45, 46], and can even occur despite fixation [47].

This sort of internally-directed attention is especially important in working memory, the short-term, capacity-limited storage and manipulation of information in an especially accessible form in service of immediate cognitive processes and goals. Working memory requires attention to highlight not only aspects of the content of perception, but also the contents of long-term memory that are relevant to the current circumstances; current models of working memory use attention to manage interactions with long-term memory by controlling what representations are maintained. Indeed, [48–51] has created an influential model that describes working memory as the selective activation of portions of long-term memory by capacity-limited attention, while [52, 53] consider working memory to have several specialized components, many of which are at least partly concerned with accessing and storing information from long-term memory using an attention mechanism. In any event, runtime learning may give us a more precise description of what placing information in long-term memory into working memory entails.

#### 1.2.5 Executive control and task switching

Goal-driven behavior is managed by a set of functions grouped together as “executive control”, which is generally considered to be centered in the prefrontal cortex. Runtime learning suggests that one of the main functions of the central executive is to determine what concepts are relevant to the current task and to direct the summoning of exemplars of those concepts from memory to use to train an appropriate model. Executive control is often examined using task-switching experiments, in which participants must rapidly adjust to performing different tasks [54]. Task-switching studies have revealed that there is a performance cost to switching from one task to another compared to repeating the same task [55]. This cost is decreased (although not necessarily eliminated) as the amount of time between the appearance of the cue indicating the switch and the presentation of the new task increases [55–57], which supports the idea that, when possible, some form of endogenous preparation or reconfiguration occurs prior to the task shift, although it is not precisely known what exactly this reconfiguration consists of.

It is possible, at least under some circumstances, that the preparation involved in task-switching is a form of runtime learning; that is, task preparation consists of performing and learning from an endogenous, self-administered, self-supervised version of the task being prepared for. However, in general, runtime learning is intended to account not for repeatedly switching between familiar tasks over relatively short timescales, but for preparing to perform idiosyncratic and/or unfamiliar tasks by converting abstract knowledge into a directly applicable form, which requires more than the reinstatement of previously-learned and possibly still-extant rules. Regardless, the evidence for preparation in experimental task-switching settings supports the plausibility of runtime learning in general.

#### 1.2.6 Mental practice and imagery

Relatedly, work in “mental practice” also seems to indicated that the basic tenets of runtime learning are plausible. In mental practice, people consciously visualize (or otherwise imagine) stimuli and the performance of an associated task in order to prepare for actually performing the task [58–60]. While early research was tentative in giving a role to practice in the performance of physical tasks (which is the typical domain that mental practice is considered and studied in), it has since been firmly established that it can have significant impact, sometimes almost as much as overt practice [61], and may even be helpful for neurological rehabilitation [62]. The benefits of mental practice have been found in a variety of motor skills, including music performance [63] and athletics [64]. [65] developed a theory of mental practice involving the priming of connections between nodes corresponding to the various components of a task (such as muscle movements).

Mental practice has been investigated using a variety of methodologies, including positron emission tomography [66] and transcranial magnetic stimulation [67]. The findings indicate that mental practice causes physical changes in the brain associated with neuroplasticity, including muscle representation in cortex [68], synaptic connectivity [67], and activity levels [66]. Presumably, if mental practice can be used to train the brain in a way similar to actual physical activity, then perhaps internal exemplars can be used to train the brain in a manner similar to real, external exemplars.

The principles underlying runtime learning may overlap with mental practice (and mental imagery) to some degree, although it differs in that the tasks are not necessarily familiar ones, and it is not necessarily a conscious process, nor is it primarily intended to account to performance in motor skills, as mental practice usually is (although of course it could be used for such purposes). Perhaps most importantly, runtime learning explicitly draws an analogy between the internal training process and conventional external training, which incidentally gives a mechanism by which mental practice itself might conceivably also work. In any event, the effects of mental practice and preparation in task-switching seems to render our proposal plausible.

#### 1.2.7 Active learning

A key conjecture of the runtime-learning hypothesis is that people learn from “high quality” data items. An important way in which people can obtain high-quality data is through active or self-directed learning [69–71]. In active learning, people are able to choose their own probes of the environment so as to, for example, receive data that maximizes the amount of new information they obtain or that minimizes their uncertainty about a task-relevant concept. Because people choose which data items to receive from the environment, learning can be rapid and efficient.

Like active learning, runtime learning supposes the identification of items that are especially valuable for learning a particular task. Many of the stimuli that would be selected by active learning would presumably be valuable for runtime learning. Unlike active learning, however, these valuable items are summoned from memory instead of found in the environment. Also unlike active learning, runtime learning uses these valuable examples to learn to perform tasks without external training, rather than to make external training more efficient.

### 1.3 Runtime learning in context

We now describe how runtime learning can be combined with earlier theories of learning and memory to form a more complete picture. When exemplars of a concept are encountered, the brain first stores them in a relatively concrete form (as in the fast hippocampal system, according to the complementary learning systems hypothesis [24, 25]). These exemplars may represent the instances that were actually experienced, or, in line with evidence of abstraction in the hippocampus, may be somewhat abstracted (as in the multiple cluster prototypes of some exemplar models). In the short term, these stored representations can be used more or less as-is for processes such as recognition involving a recently-learned concept, including using them as training data for runtime learning.

In the long term, the representations may be consolidated into a more abstract form suitable for storage (presumably in the neocortex). Depending on the circumstances, this abstraction may take one of several forms. Again, it may take the form of cluster prototypes, as in exemplar models such as the Varying Abstraction Model [18] or the SUSTAIN model [19]. Alternatively, it may take the form of a generative model of the concept that can synthesize an arbitrary number of exemplars. This generative model may be specifically trained to output exemplars that are particularly valuable for training, as in Generative Teaching Networks [15].

When a novel task involving familiar concepts is encountered or expected, the relevant conceptual memory is “deconsolidated” by either summoning the corresponding stored prototype(s) or synthesizing exemplars using the appropriate generative models, then using them to rapidly train a discriminative model for the task. For more efficient learning, the discriminative model may be initialized with parameters primed for tasks of a similar type (as in meta-learning).

## 2 Methods

### 2.1 Ethics statement

All behavioral experiments with human participants (1, 2, and 3) were approved by the Research Subjects Review Board of the University of Rochester. Experiments were administered using the world wide web, and participants gave their consent by clicking on a button.

### 2.2 Computer simulations

All computer simulation results that we report are based on an image classification task. We used images and class labels from the CIFAR-10 data set [72]. The original dataset contains 60,000 color images (each image is 32 × 32 pixels) in ten classes (6,000 images per class; the ten classes are: airplane, automobile, bird, cat, deer, dog, frog, horse, ship, and truck.). The 60,000 images are divided into 50,000 training images and 10,000 test images.

The results from a ResNet-50 classifier [73] are reported below. A ResNet model won the 2015 ImageNet Large Scale Visual Recognition Challenge [74], and ResNet models are common classifiers in the ML literature. Our ResNet-50 model was pretrained on the ImageNet dataset. However, the model was then modified to accommodate CIFAR-10 images (which are significantly smaller than ImageNet images). It was also modified by replacing the final output layer of 1000 units (suitable for ImageNet) with a new output layer of 10 units (suitable for CIFAR-10). When training ResNet-50 on our training sets, the weights of the final layer of the model were initialized randomly. All other weights were initialized to ImageNet pretrained values. The model was trained for 1850 epochs using the Adam optimizer and the cross-entropy loss function. The learning rate was initialized to 0.01, and then decreased by 90% after each 800 epochs. The remaining parameters of the Adam optimizer, *β*_1_, *β*_2_, and *ϵ*, were set to 0.9, 0.999, and 10^−8^, respectively.

### 2.3 Experiment 1

#### 2.3.1 Subjects

The experimental study was approved by the institutional review board at the University of Rochester. 141 participants took part in the experiment over the world wide web via the Amazon Mechanical Turk (MTurk) crowd-sourcing marketplace. Interfacing with MTurk was facilitated through the use of the psiTurk programming platform [75]. psiTurk was configured so that only individuals based in the United States could participate in the experiment. Participants stated that they were at least 18 years old and gave written consent electronically. It took approximately 15–20 minutes to complete the experiment, and each participant received $2.50 for their participation. The experiment consisted of practice and experimental phases (see below), and bonuses of $0.30, $0.40, and $0.50 were awarded for 70%, 80%, and 90% accuracy during the experimental phase.

#### 2.3.2 Stimuli

The experiment used stimuli from the MNIST dataset. Four digits (1, 2, 4, and 7) were used during the practice phase (2 instances of each digit), and a different four digits (3, 5, 6, and 8) were used during the experimental phase (20 instances of each digit for each participant). The latter digits were chosen based on examination of published confusion matrices for MNIST recognition systems [76–78], as we wanted stimuli that participants would have a relatively high chance of misclassifying. Participants were randomly assigned to one of four groups, where each group saw different sets of instances of each digit. Thus, there were a total of 4 groups × 4 digits × 20 instances per digit per group = 320 instances of digits used in the experiment.

#### 2.3.3 Procedure

During the practice phase of the experiment, on each trial, subjects viewed a display which showed a fixation cross for 500ms, followed by a random noise mask consisting of black and white pixels for 100ms, then one of the handwritten digits for 100ms, and finally another noise mask for 100ms. Following this stimulus presentation, participants were asked to press the key corresponding to the identity of the digit (e.g., the “1” key in response to the display of a 1). Importantly, the task was often challenging, due to the short display time of a digit and the presence of the forward and backward noise masks. Each participant completed 8 trials during this phase. Digit instances were used in random order, but each instance was guaranteed to appear exactly once. Responses and response times were recorded. Data from the practice phase were not analyzed. This phase served to familiarize participants with the experimental procedure and general characteristics of the stimuli.

After the practice phase, participants completed the experimental phase. Procedurally, experimental trials were identical to practice trials (though, of course, they used different digits). As in the practice phase, digit instances were used in random order, but this time each instance was guaranteed to appear exactly *two* times, for a total of 160 trials (4 digits × 20 instances per digit × 2 displays per instance). To help maintain interest and attention, every twenty trials participants were informed of how many correct responses they made during those trials (although they were not told which trials they had answered correctly).

### 2.4 Experiment 2

#### 2.4.1 Participants

This web-based experimental study was conducted in a manner similar to Experiment 1. 123 participants took part in the experiment. The experiment took approximately 30–45 minutes to complete, and each participant received $6.00 for their participation. The experiment consisted of training and test phases (see below), and bonuses of $0.60, $0.80, and $1.00 were awarded for 70%, 80%, and 90% accuracy during the test phase.

#### 2.4.2 Stimuli

The experiment used stimuli from the Devanagari Handwritten Character Dataset [79], an MNIST-like dataset consisting of handwritten exemplars of characters of Devanagari, a Brahmic script used to write languages such as Hindi. It is assumed that these characters are generally unfamiliar to participants in the United States, and thus can be considered “novel” for our purposes. Four characters (known as “tabala”, “waw”, “patalosaw”, and “ha”) were used. Each character was arbitrarily associated with the Arabic digit 1, 2, 3, or 4 (e.g., all “tabala” characters were associated with 1). Four sets of stimuli were constructed, each consisting of 20 randomly-selected exemplars of each character (for a total of 80 exemplars in each set). Thus, there were a total of 4 groups × 4 characters × 20 instances per character per group = 320 instances of characters used in the experiment. Participants were randomly assigned to see one of the four groups during the training phase, and one of the remaining three groups during the test phase, and during both phases each exemplar in the group was seen twice.

#### 2.4.3 Procedure

During the training phase of the experiment, on each trial, subjects viewed a display which showed a fixation cross for 500ms, followed by a random binary noise mask for 250ms, then one of the handwritten characters for 500ms, and finally another noise mask for 250ms. Following this stimulus presentation, participants were asked to press the key corresponding to the identity of the character. After making their response, participants viewed a screen which informed them of the correct answer. Using this feedback, subjects could learn to perform the classification task—via trial and error—with these novel stimuli. Importantly, the task was often challenging, due to the short display time of a character, the presence of the forward and backward noise masks, and the novelty of the stimuli. Each participant completed 160 trials during this training phase. Character instances were used in random order, but each instance was guaranteed to appear exactly twice. Responses and response times were recorded. Data from the training phase were not analyzed.

After the training phase, participants completed the test phase. Procedurally, test trials were identical to training trials, but used a different group of exemplars. In addition, mask and stimulus presentation times were shortened to 100ms each. As in the training phase, character instances were used in random order, and each instance was guaranteed to appear exactly two times, for a total of 160 trials (4 characters × 20 instances per character × 2 displays per instance). To help maintain interest and attention, every twenty trials participants were informed of how many correct responses they made during those trials (although they were not told which trials they had answered correctly).

### 2.5 Experiment 3

#### 2.5.1 Participants

This web-based experiment was conducted in a manner similar to that of Experiments 1 and 2. 88 participants took part in the experiment. The experiment took approximately 25–35 minutes to complete, and each participant received $4.50 for their participation. The experiment consisted of training and test phases (see below), and bonuses of $0.50, $0.60, and $0.70 were awarded for either 50%, 60%, and 70% accuracy or 40%, 50%, and 60% accuracy, for participants who were trained with the good and bad datasets, respectively (see below), during the test phase.

#### 2.5.2 Stimuli and procedure

The stimuli and procedure were nearly the same as in Experiment 2. However, participants were trained with either the “good” set (39 participants) or the “bad” set (49 participants) identified in Experiment 2. Consequently, training lasted 40 trials (4 characters × 5 instances per character × 2 displays per instance). Stimuli used during testing were randomly selected with the constraint that test items could not be members of the good or bad training sets. All exemplars from the previous experiment that were not part of either the good or bad training sets were retained, with the substitutions for the remainder selected randomly; two substitutes ended up being used twice, for a total of 318 different test exemplars.

#### 2.5.3 Experiment analysis

The preceding three experiments were analyzed with the use of a deep neural network (DNN). The DNN was a convolutional network with a convolutional layer with 6 filters of size 5 × 5, a stride of 1, and rectified linear units, followed by a max pooling layer with 2 × 2 filters and 50% dropout during training, followed by another convolutional layer with 16 filters of size 5 × 5, a stride of 1, and rectified linear units, then another max pooling layer with 2 × 2 filters and 50% dropout during training, followed by a densely connected layer with 128 rectified linear units, a densely connected layer with 64 rectified linear units, and finally 4 softmax output units. The weights were randomly initialized by sampling from a He normal distribution [73] for all but the last layer, which was initialized with a Glorot uniform distribution [80]. All layers but the last had 50% dropout. The network was trained using the Adam optimization algorithm [81] for 64 epochs with a batch size of 64, an alpha of 0.005, and a categorical crossentropy loss. (Note that we are not making any particular claims about the architecture or algorithm that is being runtime trained. As we noted earlier, many algorithms for few-shot learning, such as matching networks, are compatible with runtime learning; we have used basic convolutional networks for simplicity. In fact, it would not surprise us if it is ultimately discovered that different tasks use different architectures.)

## 3 Results and analysis

### 3.1 Computer simulations

In this section, we begin to evaluate the runtime-learning hypothesis by examining if and under what circumstances neural networks can efficiently learn from a limited number of training items. In particular, we investigate whether the training items identified using human data are effective, and if and when they are better than randomly selected training items. (However, we are not examining what methods the *brain* might use to identify such instances.)

#### 3.1.1 Datasets based on human responses

This subsection reports the results when the ResNet-50 classifier was trained on datasets in which items were selected based on human experimental data. [82] conducted an experiment in which subjects were asked to classify images from the CIFAR-10 test set. In total, these researchers collected more than 500,000 responses distributed over all 10,000 test images. Some images were classified with high confidence, meaning that subjects were relatively consistent in the class labels assigned to those images. In contrast, other images were classified with low confidence, meaning that different subjects tended to respond to these images with different class labels.

Using this database, [82] found that training DNNs with a training set in which each item is weighted by the inverse of the entropy (a measure of uncertainty) of the human response distribution for that item improved the performance of the trained model. Our experiment is similar in nature, but distinct: while in their experiment the exemplars varied in importance, they were still all included in the training set. Since we are interested in speeding up the training process, we want to examine whether we can use the human behavioral data to efficiently reduce the amount of training required. It is plausible that the extra information included in the weighting labels used by [82] improves performance while, at the same time, the high-confidence exemplars, by themselves, are inadequate for training to a high level of performance. Here we assess this possibility.

We evaluated the confidence with which an image was classified by calculating the entropy of the probability distribution over classes produced by subjects. High-confidence images were those with low entropy distributions, whereas low-confidence images were those with high entropy distributions. High-quality, low-quality, and random training sets, denoted $D_{HQ}$, $D_{LQ}$, and $D_{R}$, respectively, were created as follows. $D_{HQ}$ and $D_{LQ}$, each with 1000 images, were created by selecting the 100 highest-confidence and lowest-confidence images from each class, respectively. $D_{R}$ was created by randomly selecting 100 images from each class from the CIFAR-10 training set. An 8000-image test set, denoted $D_{T}$, was also formed, consisting of the 800 images from each class from the CIFAR-10 test set that were not part of $D_{HQ}$ or $D_{LQ}$.

**Fig 1:**
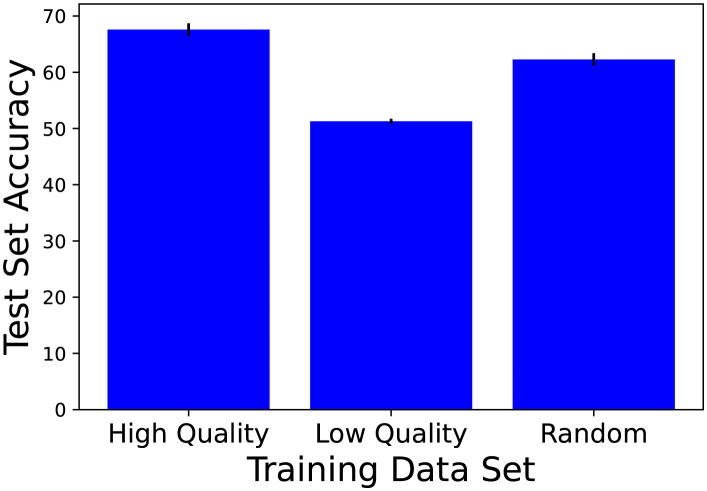
Computer simulation results. Average accuracy (percent correct) over five runs on test set $D_{T}$ after ResNet-50 was trained on $D_{HQ}$, $D_{LQ}$, or $D_{R}$. Error bars show the standard errors of the means.

Fig 1 shows the average accuracy (percent correct) on test set $D_{T}$ after ResNet-50 was trained on $D_{HQ}$, $D_{LQ}$, or $D_{R}$. (ResNet-50 was also trained and tested on the entire CIFAR-10 dataset (50,000 training items; 10,000 test items) and achieved an average test-set accuracy of 95.65% correct.) Relative to training with random items, training with high-quality items led to 9% better test performance, whereas training with low-quality items led to 18% worse performance.

The results in this section demonstrate that a learning system can more efficiently learn from a small dataset of high-quality items than from an equal-sized set of random items. Consequently, when no currently suitable network is available—and thus extemporaneously learning from a small dataset is the best option—it is sensible for an agent to learn solely from high-quality data items. This might be considered a surprising result, as in the field of ML it is generally thought that learning will be best when distributions over training and test items are the same, so that the training items are representative of test items. Using that logic, one might expect training with random dataset $D_{R}$ to lead to the best test performance, but we did not find that to be the case. Instead, we found training with high-quality dataset $D_{HQ}$ to be best despite the mismatch in distributions over training and test items. We examine this question more thoroughly in S2 Appendix. Finally, we found that human subjects’ responses in a classification experiment can be used to identify high-quality and low-quality data items.

### 3.2 Interim discussion

So far, we have proposed a relationship between learning and memory, namely that people often rapidly learn to perform tasks from small datasets of high-quality items obtained from memory, especially when suitable previously-trained neural networks are unavailable. The plausibility of this runtime-learning hypothesis was evaluated using DNNs and an image classification task. It was found that training with high-quality items led to better test performance than training with random or low-quality items when item quality was based on human subjects’ responses in an experimental study.

As described in S2 Appendix, similar results were found when item quality was based on DNN performance. Moreover, it was found that the advantages of training with high-quality items were particularly significant when training sets were very small. We conjectured that, despite the concomitant mismatch between distributions over training and test items, training with high-quality items is sensible due to the fact that this mismatch is likely to be moderate in magnitude because high-quality training items tend to be near cluster centers, and thus help learning systems quickly learn cluster locations. Additional results showed that high-quality items helped a DNN learn to nonlinearly map images to appropriate clusters such that the high-quality items were near cluster centers in an abstract feature space (see S2 Appendix).

Critically, training examples identified using human behavioral data facilitated DNN learning more than other examples. This result is at least consistent with the hypothesis that humans do indeed perform runtime learning. This interpretation allows us to conjecture why subjects’ responses were useful for selecting training items. Presumably, the runtime-trained networks that people use to perform a task respond most confidently to experimental test items that resemble the valuable examples used during runtime learning. Thus, those confidently-identified test items are probably valuable examples as well, explaining why subjects’ responses in a behavioral experiment are useful for selecting training items that facilitate learning. The remainder of the paper explores this idea in depth.

It should be noted that the runtime learning hypothesis is an inherently difficult one to test, as it requires researchers to identify internal mental processes based on differences in behavior that the hypothesis itself states are small and underdetermined. Nevertheless, we believe that the hypothesis is an important one, and that the evidence we present is compelling enough to provide significant support and encourage future investigation (both by us and by others).

### 3.3 Experiment 1

The use of symbolic reasoning algorithms such as semantic networks to select concepts is a well-established part of classical artificial intelligence that has been demonstrated many times; therefore, we focus on the other main aspect of runtime learning, namely that humans make judgments based on extemporaneously-trained models, which must be trained quickly and therefore with a relatively limited dataset. ML researchers are actively searching for new and more effective dataset distillation algorithms. However, we wondered whether data from a behavioral experiment with people might be useful for identifying small subsets of “good” training items, meaning items that a learning system would find most useful. For instance, it may be that data items which people find easy—those they respond to quickly and accurately—are good training items for a learning system, as suggested by our results in the previous section. We also wondered if human behavioral data could be accounted for using neural networks trained using a small, curated training set.

#### 3.3.1 Analysis

**Accuracy and reaction time**. We can only expect to obtain useful data from participants who performed the task at least moderately well. Because many participants performed poorly, presumably due to its inherent perceptual difficulty, we restricted our analyses to responses from the subset of participants who responded correctly on at least 75% of the trials in the experimental phase (the task is evidently quite difficult, as this left only 98 participants).

For each participant, we took the rank of that participant’s average reaction time to each individual digit instance (across the two displays of that instance), and compared it to the average reaction time to the other instances of the same digit. Thus, for each participant, we had four sets or rankings (one for each digit), where a set consisted of the ranking of twenty instances of a digit. Using rank, as opposed to absolute reaction time, meant that we did not have to normalize reaction times across participants, and also minimized the effect of outliers.

For each participant and each instance of a digit, we noted how often the participant correctly classified the instance (either 0, 1, or 2 times), and then averaged these values across participants. To check for a relationship between accuracy and reaction time, we calculated the Spearman’s rank correlation between the average number of correct responses to an instance and the average reaction time rank of that instance, resulting in a correlation of −0.638, *p* < 1 × 10^−36^. In other words, participants responded faster to instances when the instances were more likely to be classified correctly.

**Easy/difficult instances from reaction times**. Having confirmed that reaction time correlated with accuracy, we supposed that the speed at which responses were made could be used as a measure of the ease with which an instance could be classified. For each instance, we conducted a t-test comparing all ranks of that instance (by all the participants who saw that instance) to all the ranks of all the remaining instances of the same digit. We thus identified instances that had a significantly lower or higher average rank compared to other instances.

**Fig 2:**
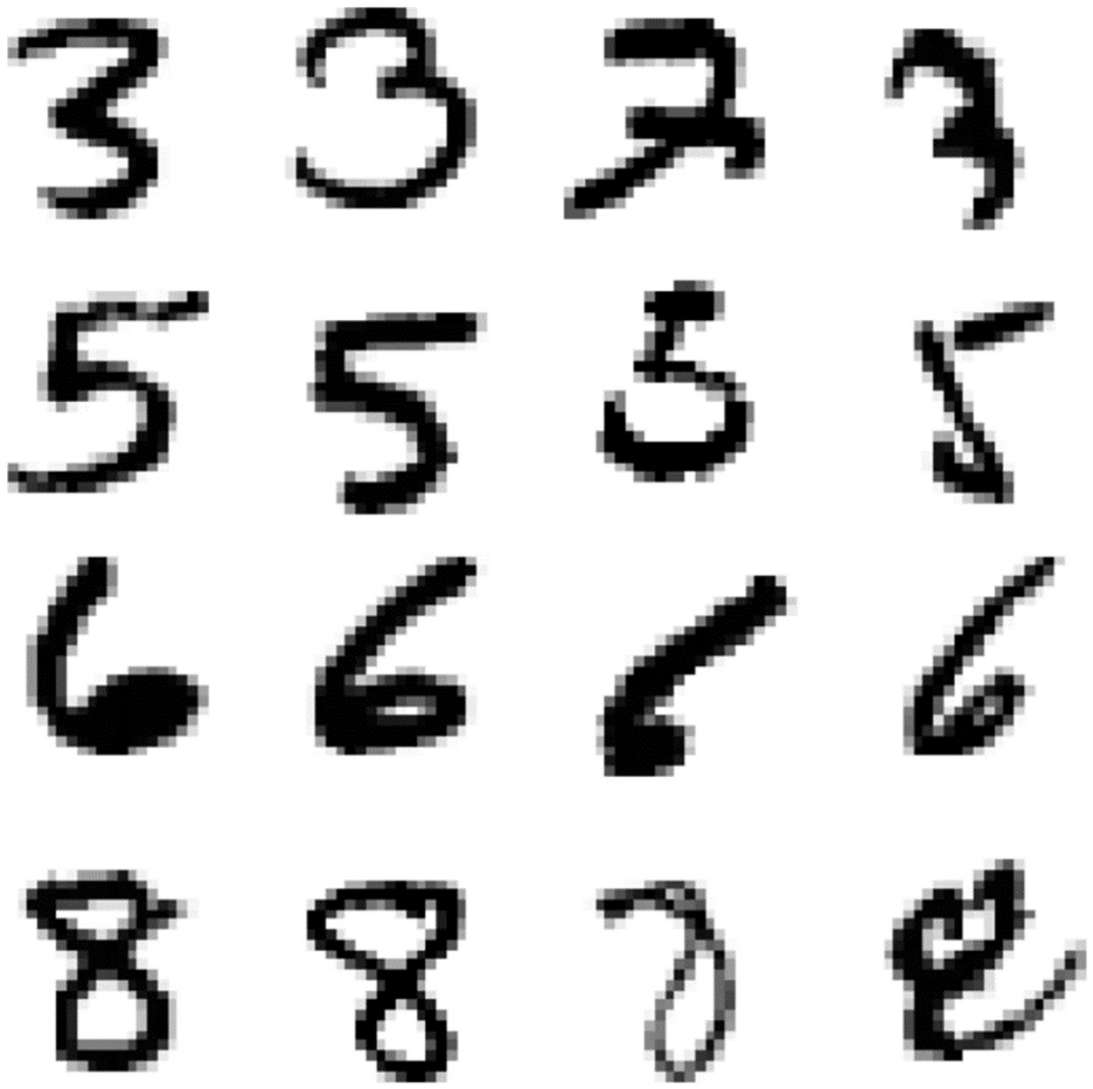
Easy and difficult MNIST instances. The left two columns show “easy” instances of Arabic digits (i.e., instances with significantly below average reaction time ranks), whereas the right two columns show “difficult” instances.

From visual inspection, the results are reasonable and intuitive. Fig 2 shows some representative examples of easy instances (left two columns) and difficult instances (right two columns). Easy instances clearly exhibited the salient features of the digit, whereas difficult instances often possessed deformities.

#### 3.3.2 Creating small training sets

Assuming easy instances are relatively typical of their categories, whereas difficult instances are atypical, we used the reaction time ranks from our behavioral experiment to create small training datasets for machine learning systems. The “bad” training set consisted of the twenty instances, five from each digit, with the most significantly (most *p* < .05, all *p* < .07) higher average reaction time rank than the other instances. The “good” training set consisted of the twenty instances, five from each digit, with the most significantly (most *p* < .05, all *p* < .07) lower average reaction time rank. (We disqualified and excluded one otherwise significant “5” exemplar because it was never correctly identified by any participant due to its close resemblance to a “6”. The speed of its reactions were therefore not due to its typicality or ease of identification as a “5”.) Finally, we also constructed several “random” training sets of randomly chosen instances (subject to the constraint that, as with the other two sets, they each contain five instances from each digit).

However, even with the most valuable 20 training items, it is unreasonable to expect a training set of this size to be able to train a neural network (or other classifier) to do anything besides overfit. For this reason, we used data augmentation to increase the sizes of the sets. In this augmentation, the set of black pixels in each instance were shifted left or right and/or up or down one pixel, and/or rotated left or right by 10 degrees to produce additional instances of the same digit. For each training set, this resulted in 540 data items (9 possible shifts × 3 possible rotations × 20 initial instances). In addition to the good, bad, and random sets, we also applied this procedure to the “full set”, consisting of all 320 instances used in the behavioral experiment, so that the resulting training set (with 9 × 3 × 320 = 8640 items) could be used to establish a baseline level of performance to compare with the performances achieved following training with smaller sets. (We chose this baseline, instead of using all 23,321 relevant exemplars in the MNIST database, because we wanted to limit the comparison only to exemplars that analysis of our experimental data could possibly have identified as valuable.) These training sets were used to train a deep convolutional network, described earlier under Methods.

**Fig 3:**
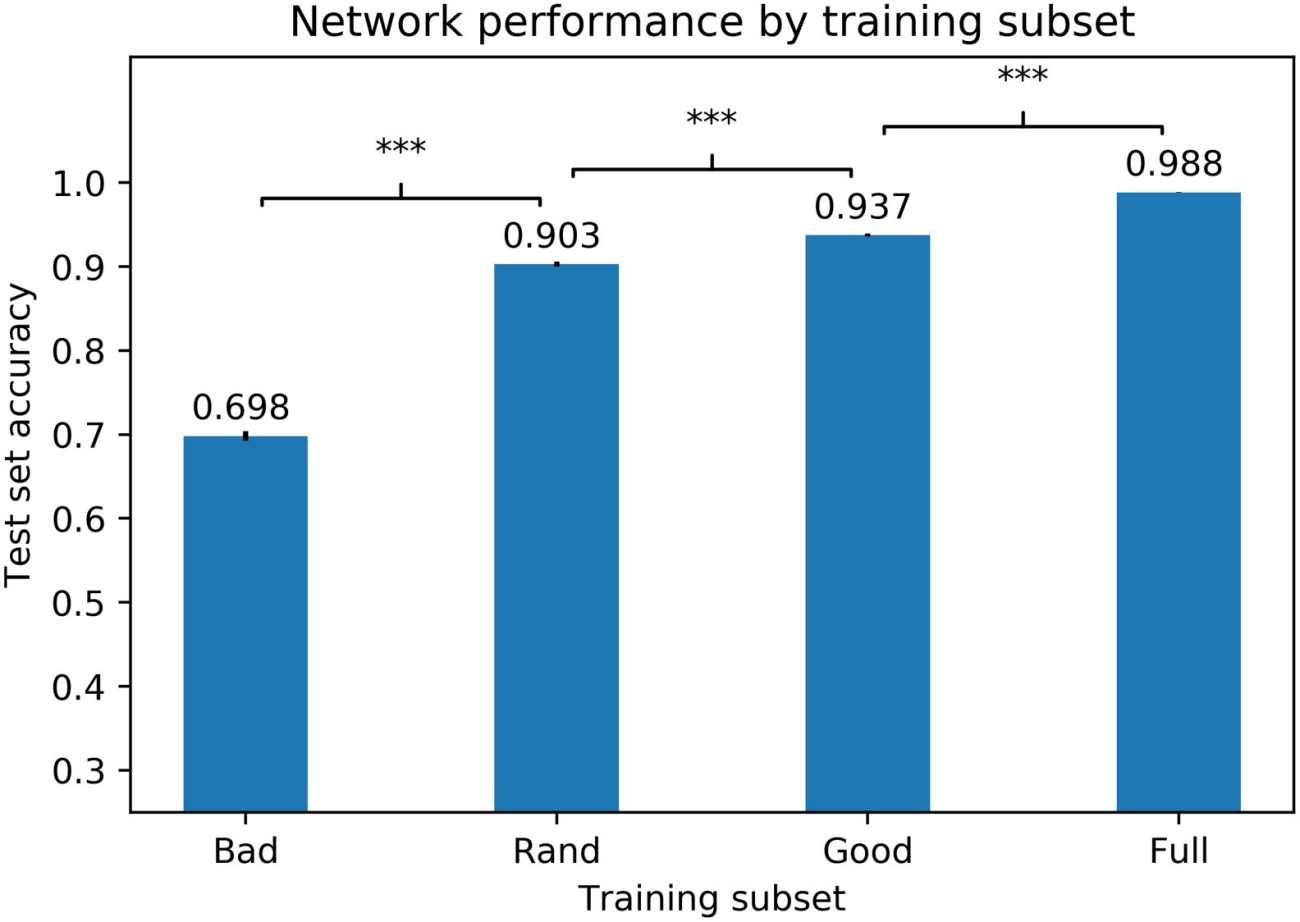
DNN accuracy by training subset for MNIST. Average test-set accuracies for neural networks trained on bad, random, good, and full training sets (numerical values are shown above the bars), based on training one hundred randomly-initialized networks on each training set. Error bars show standard errors of the means. Brackets indicate the level of significance at which pairs are different: ***: *p* < 0.001; **: *p* < 0.01; *: *p* < 0.05; n.s.: *p* > 0.05.

**Network accuracy**. After training, a network was tested on the 3,834 relevant exemplars from the MNIST test set. Each dataset was used to train one hundred randomly initialized networks. Results are shown in Fig 3. Networks trained on the bad training set performed worst, networks trained on the random sets performed better, networks trained on the good set performed even better, and networks trained on the full set performed best, with the differences between all pairs being significant (*p* < .001 in all cases). Even though the full set contained 16 times as many training items as the good set, networks trained on the full set performed only 5% better. Overall, these results suggest a connection between ease of participant classification and training value for machine learning algorithms, and establishes that a small curated dataset can be used to train neural networks to a high level of performance.

The point of this analysis is not simply that using the “good” subset is almost as effective as the full subset; rather, the most important point is that it is better than randomly selecting exemplars (and better at a high level of significance). This means that if, as we claim, our participants’ judgments must be based on a relatively limited number of exemplars, then these exemplars are especially valuable ones and therefore the logical choice to use under those circumstances. In experiment 3, we will examine whether the exemplars identified using this method are indeed especially valuable for humans. For now, we will next analyze whether our results support the notion that humans are basing their judgments on a limited training set such as this.

#### 3.3.3 Accounting for subject reaction times

In this subsection, we account for subject reaction times in our experiment by modeling reaction times with network confidences.

**Modeling reaction times with network confidences**. Because we used a softmax activation function for our network’s output units, along with a categorical crossentropy loss during training, we can interpret the activation values of these units as category probabilities. For each data item, we used the entropy of the probability distribution defined by the activation values of the output units as a measure of a network’s “confidence” (e.g., when entropy is low, the network can be considered confident in its answer). Presumably, participant reaction time similarly reflects some form of confidence in a response, with faster reaction times indicating higher confidence, so the question arises whether there is a relationship between participant reaction time to a stimulus and network confidence in its answer for the same stimulus.

However, it is not immediately obvious what the proper way to compare these two quantities is. Human response time is not solely determined by the decision process, but is also affected by other factors such as planning and executing the motor response. Thus, even if our network perfectly replicated the human decision process, we would not expect the distribution of response times and the distribution of network confidences to match exactly. But with some consideration of what we know about human reaction time, perhaps we can determine how the distributions of these quantities should be related.

Human reaction times under diverse circumstances can be accurately described by various models that treat the decision process as one of stochastic evidence accumulation toward one or more thresholds or boundaries, with prominent examples including the Drift Diffusion Model [83–85], the Leaky Competing Accumulator [86], and the Linear Ballistic Accumulator [87]. These models generally use similar psychologically relevant parameters, including latent period length (the amount of time devoted to non-decision processes that act to shift the distribution of reaction times to the right), the boundary separation (the locations of the boundaries or thresholds denoting different decisions), and the drift (the rate at which evidence supporting a particular decision is stochastically accumulated).

Importantly, of these parameters, only the drift is influenced by the individual stimulus presented in a particular trial, with high drifts being produced by the presence of high-quality evidence—the rate at which evidence can be gathered is obviously limited by the amount of evidence present to begin with. In contrast, the boundary location is preselected, and the nondecision component is presumably fixed. Therefore, any systematic effect the individual exemplars have on the distribution of reaction times (and by extension, the summary statistics of that distribution) is due to their effect on drift, and we can use the mean reaction times to a particular stimulus as a proxy for the drift induced by that stimulus, with low means indicating high drift rates.

Turning now to the neural networks, they obviously have no nondecision component to their responses, and due to the softmax activation function that determines their output, they have nothing corresponding to boundary separation either. Furthermore, trained neural networks, as least as they are commonly used, are deterministic and have no stochastic component. However, the values in the output units can be readily interpreted as representing the relative amount of evidence for each possibility, and the entropy of these units can be seen as quantifying how concentrated the evidence is. Intuitively, this corresponds to the drift rate in the diffusion model: low entropy corresponds to high confidence and high drift rate.

**Fig 4:**
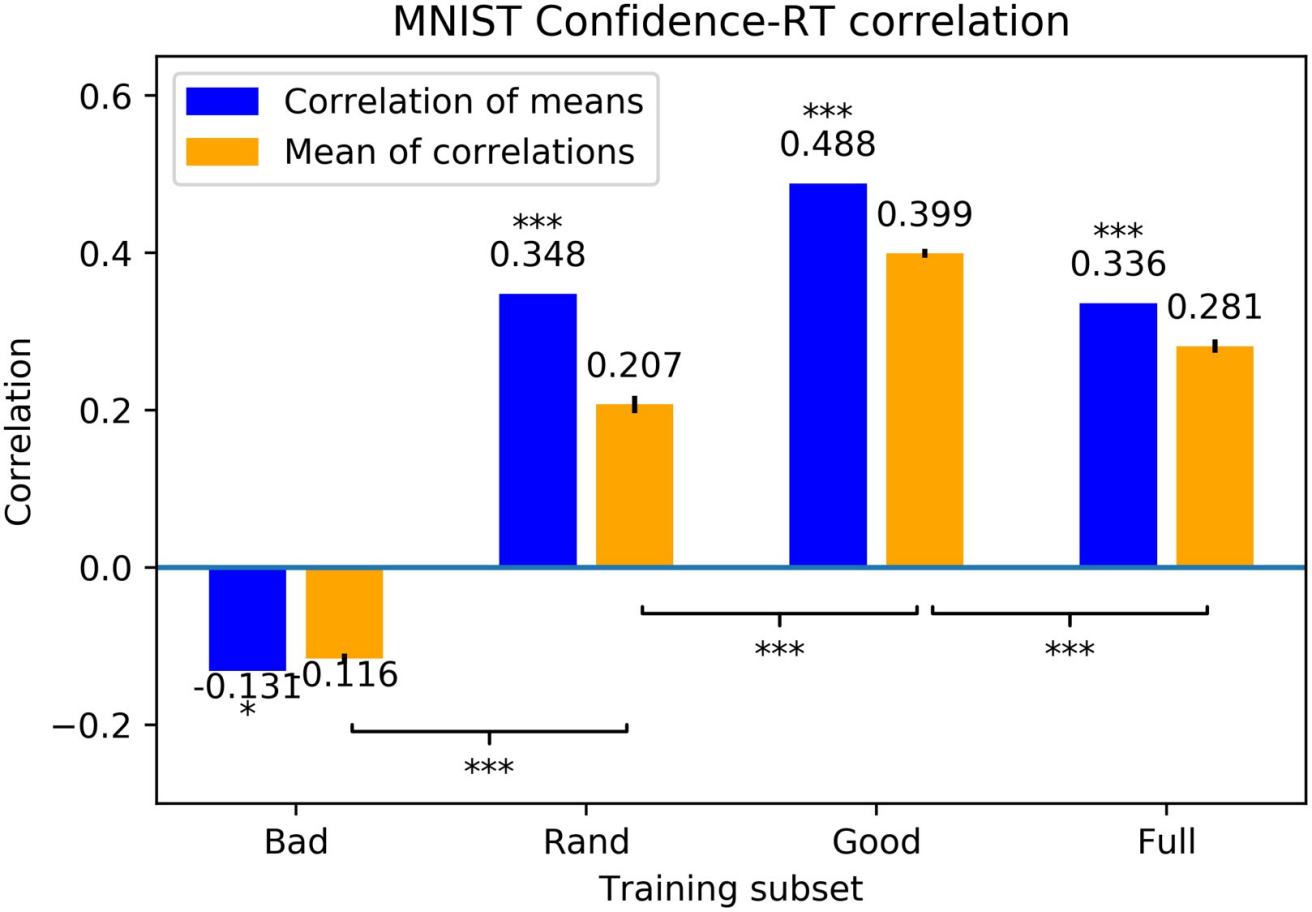
Correlation between human reaction time and DNN confidence for MNIST. Spearman rank correlation coefficients between the mean of networks’ confidences and the mean of normalized participant reaction times, based on training one hundred randomly-initialized networks on each training set. The left, blue bar is the correlation between the mean network confidence for each exemplar and mean human RT for the same exemplar; the right, orange bar is the mean of the correlations between each network’s confidence and mean human RT, with error bars representing standard errors of the means. Asterisks above bars indicate the level at which the correlation was significantly different from zero; asterisks on brackets indicate the level at which pairs of sets of correlations are different from each other: ***: *p* < 0.001; **: *p* < 0.01; *: *p* < 0.05; n.s.: *p* > 0.05.

Therefore, to the extent that the neural networks reflect the human decision process, we would expect to see a correlation between the mean reaction time to a particular exemplar and the entropy of neural network responses to that exemplar. We thus found the Spearman’s correlation coefficients between the mean of human reaction times (which were normalized such that each participant’s reaction times to all the exemplars they saw summed to 1) to an exemplar and the mean confidence (similarly normalized) induced by the same exemplar in 100 neural networks set up in the same way as before and trained on each of the good, bad, random, and full training sets. We also found the Spearman’s correlation coefficients between the mean of human reaction times and the confidences of *each* network. The results, shown in Fig 4 (left graph), indicate a moderately strong and highly significant correlation for networks trained on the good set, lesser correlations for random sets and the full dataset, and a negative correlation for networks trained on the bad set. (Some readers may be surprised by the negative correlation exhibited by the networks trained on the bad subset. We believe this negative correlation is due to the networks trained on the bad set learning to rely on different, possibly less useful features than humans and networks trained on the good set. These features may be just as present, or even more so, in exemplars that the other networks are less confident in. Thus the networks trained on the bad subset may have confidence in different exemplars, and thus negatively correlated. To put it another way, if “bad” exemplars are far from the true cluster centers, then models trained on them will be most confident on exemplars that are also far from the cluster centers, the opposite of the trend seen in humans and “good”-trained models, which are confident in exemplars close to the cluster centers.) Furthermore, the individual correlations produced by the good set were different from those of other sets at a higher level of significance. Thus, based on both the direction and magnitude of the correlation, networks trained on the good training set provide the best account of participants’ reaction times in our experiment. This provides intriguing evidence that, consistent with the runtime-learning hypothesis, participants in our experiment may have categorized stimuli based on a small set of good exemplars. In S3 Appendix, we further analyze this data using a drift-diffusion model of human reaction times, with similar results.

#### 3.3.4 Discussion

Our results demonstrate a congruence between the ease with which an exemplar of a category is classified by participants and the usefulness of that exemplar in training a DNN to recognize that category. We explain this by postulating that participants similarly complete the task by training a network using especially valuable examples, and the most easily identified test examples are valuable because they are similar to these hypothetical valuable training set examples, and are thus valuable themselves. This explanation is further supported by relatively large correlations of the confidence values of networks trained on the valuable examples with both participant reaction times and fitted drift rates.

The high correlations associated with the good dataset cannot be explained merely as due entirely to correlations involving the specific exemplars used to train the neural networks; these correlations seem much too large to be produced by only these few exemplars. A reasonable conclusion is that these correlations exist because training with the good dataset yields neural networks with similar “reaction times” to our human participants across a relatively large range of the probability distribution over the stimulus space. That is, training on a small but curated dataset produces the best match in reaction times *throughout* the distribution. In contrast, a model that was simply overfit to the training set would not be expected to be so correlated with the human data.

### 3.4 Experiment 2

If, as we hypothesize, preparing to perform a task involves summoning particularly valuable exemplars for internal training, part of the process of learning is the identification and storage of those exemplars for later retrieval. One possible (although not necessary) consequence of this would be that even when people have the opportunity to learn from many environmentally-provided examples, their performance on a task is reflective of training on a limited subset of those examples, namely the most valuable ones (or valuable abstractions, as in the exemplar models that learn cluster prototypes). In other words, people would be immediately putting their learning of valuable examples into practice by giving those examples exclusive importance during the concurrent training process. To examine this possibility, we conducted an experiment similar to Experiment 1, but which required participants to first learn to perform the classification task on unfamiliar characters from the Devanagari alphabet, instead of relying on prior knowledge.

#### 3.4.1 Analysis

**Fig 5:**
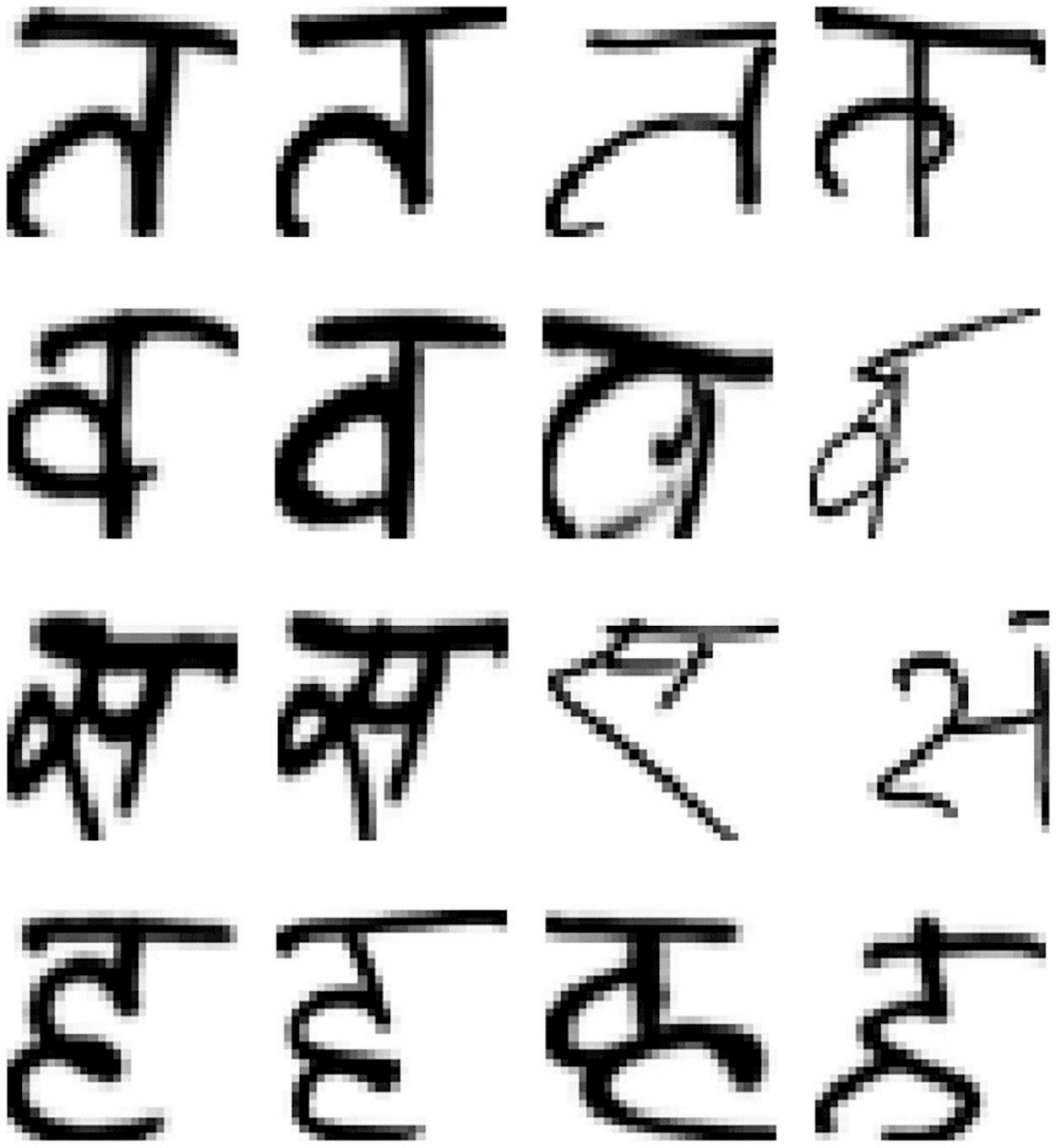
Easy and difficult Devanagari instances. The left two columns show “easy” instances of Devanagari characters (i.e., instances with significantly below average reaction time ranks), whereas the right two columns show “difficult” instances.

Because not all participants adequately learned the character classification task, we restricted our analyses to responses from the subset of participants who responded correctly on at least 75% of the test trials (leaving 54 participants). We subjected these participants’ test data to the same rank-based analysis as used in Experiment 1, thereby identifying exemplars that participants were particularly (all *p* < .08, most *p* < .05) quick or slow to categorize (see Fig 5 for examples). This analysis allowed us to create good and bad training sets. As before, each set consisted of 5 instances of each character.

**Fig 6:**
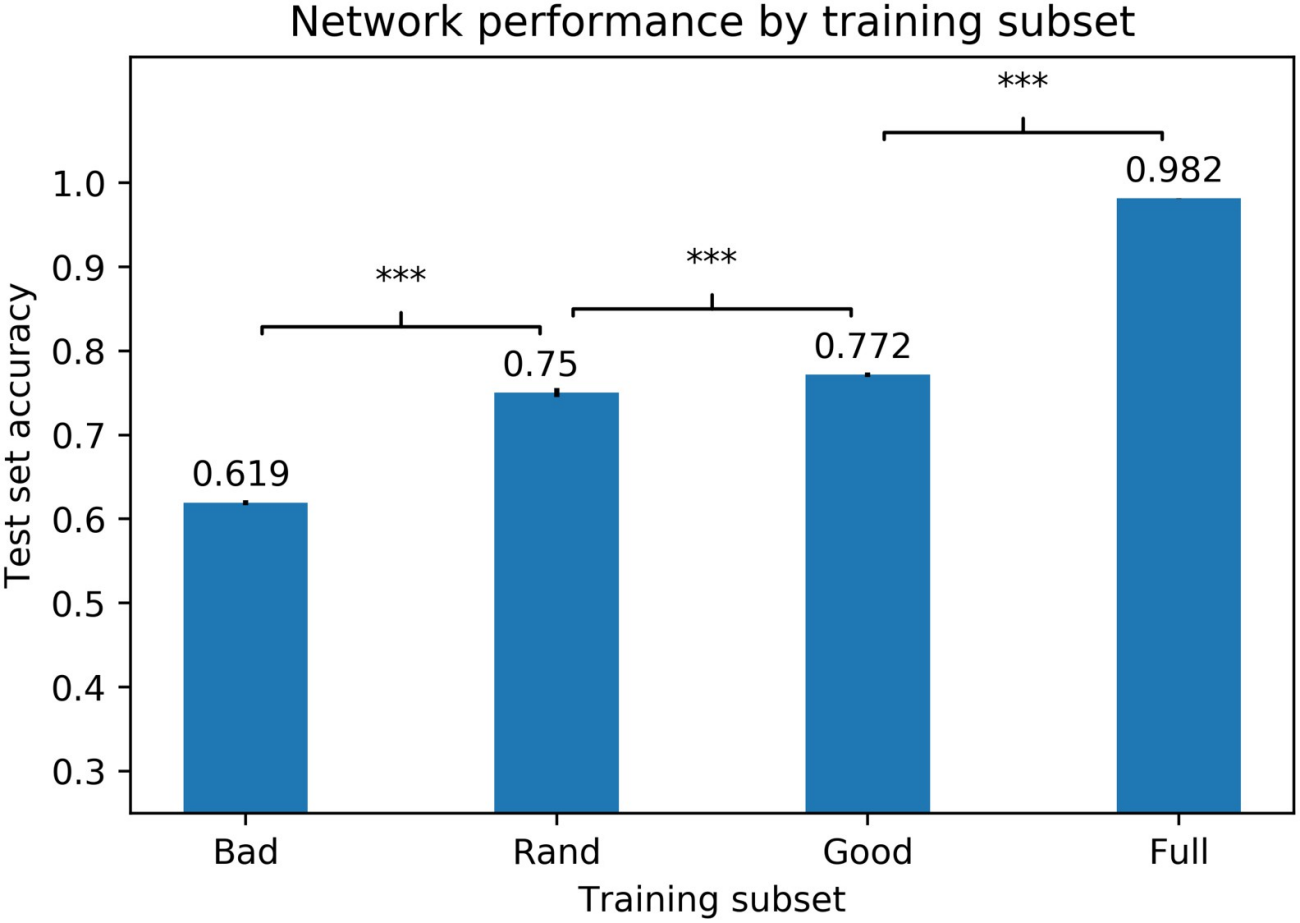
DNN accuracy by training subset for Devanagari. Average test-set accuracies for neural networks trained on bad, random, good, and full training sets (with data augmentation), based on training one hundred randomly-initialized networks on each training set. Error bars show standard errors of the means. Brackets indicate the level of significance at which pairs are different: ***: *p* < 0.001; **: *p* < 0.01; *: *p* < 0.05; n.s.: *p* > 0.05.

As in Experiment 1, we trained neural networks with good and bad training sets, with random training sets (each set contained 5 randomly selected instances of each character), and with a full training set (consisting of all 80 instances of each character used in the experiment). In each training condition, one hundred randomly-initialized DNNs were trained, and data augmentation was used as described above. Following training, networks were tested on the relevant items from the Devanagari Handwritten Character Dataset test set. Test-set accuracies are shown in Fig 6. As expected, network performance was worst with the bad training set, better with the random set, and even better with the good set, with the differences between all pairs significant at *p* < .001.

**Fig 7:**
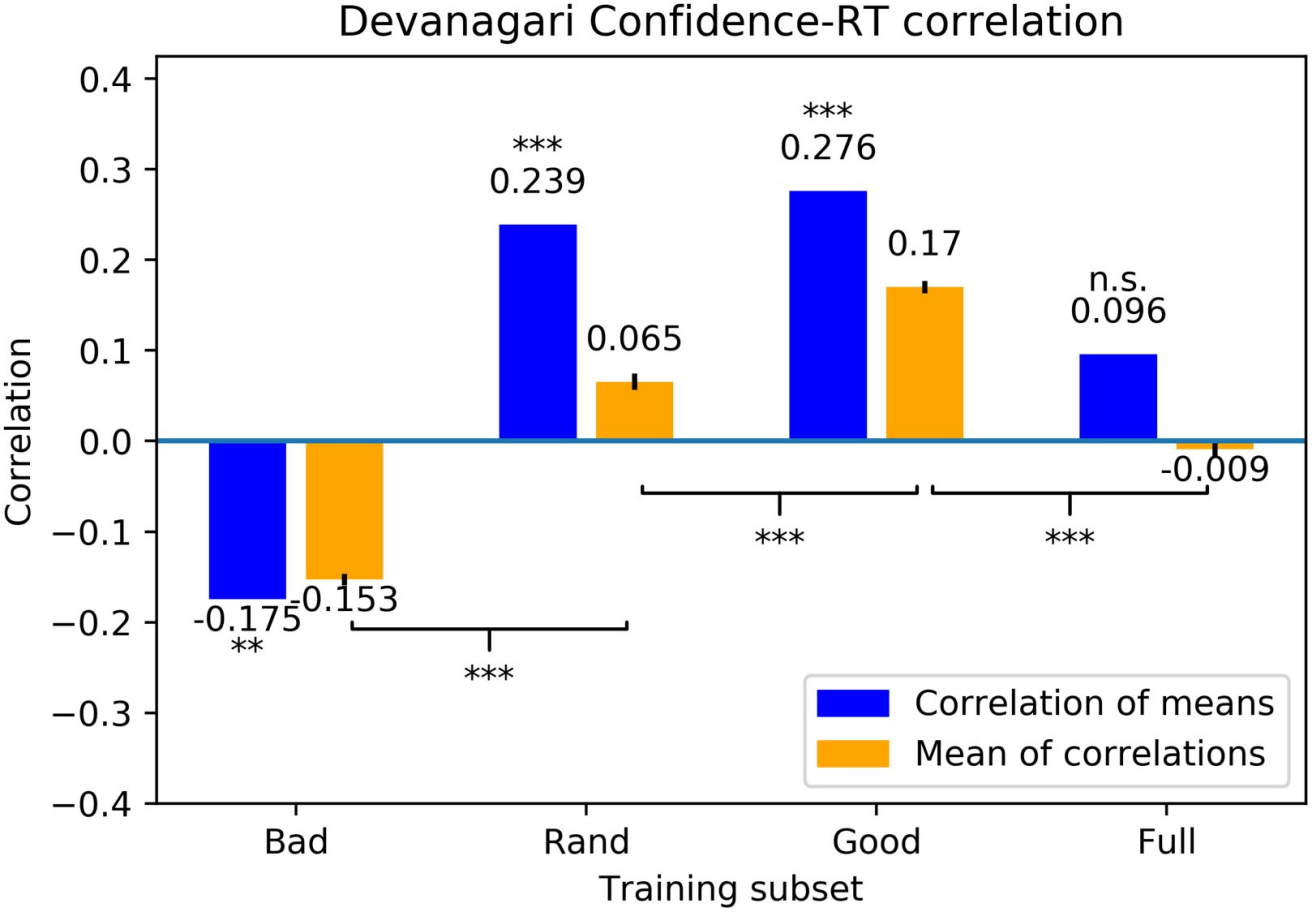
Correlation between human reaction time and DNN confidence for Devanagari. Average Spearman rank correlation coefficients between networks’ mean normalized confidences and participant’s mean normalized reaction times based on training one hundred randomly-initialized networks on each training set. The left, blue bar is the correlation between the mean network confidence for each exemplar and mean human RT for the same exemplar; the right, orange bar is the mean of the correlations between each network’s confidence and human RT, with error bars representing standard error of the means. Asterisks above bars indicate the level at which the correlation was significantly different from zero; asterisks on brackets indicate the level at which pairs of sets of correlations are different from each other: ***: *p* < 0.001; **: *p* < 0.01; *: *p* < 0.05; n.s.: *p* > 0.05.

We also computed correlations between network confidences and participants’ mean normalized reaction times (as in Experiment 1). Correlation coefficients are shown in Fig 7. Crucially, DNNs trained on the good training set once again provided the best account of participants’ reaction times in magnitude, direction, and significance level. Analysis based on a drift diffusion model is presented in S3 Appendix. We also present a number of variations of our analysis in S4 Appendix with the aim of reducing the possibility that our results are due to some sort of artifact.

#### 3.4.2 Discussion

The data from Experiment 2 exhibit the same general patterns of results as those from Experiment 1 in both our performance-based and confidence-based analyses. However, the magnitude of the performance effect is lower than with the MNIST digits, which is what we would expect. Our participants in Experiment 1 have been using Arabic digits for years, and although they would have to adapt to our specific task, they already have a very good sense of what “good” examples of each of those digits are. On the other hand, participants in Experiment 2 are identifying valuable examples based on very limited training, so it is not surprising that those exemplars, while more effective than average, are not the best possible. Thus, while the good subset usually (and significantly) outperforms the random subset, occasionally the random subset will by chance consist of exemplars that are even more effective, reducing the difference between the performance produced by the good and random subsets. This effect may be further strengthened because, as can be seen from the overall level of performance, the Devanagari characters are more difficult to learn than the Arabic digits. At the same time, the correlation effect remains relatively strong, indicating that the good subset really does capture something important about the learning processes underlying our participants’ performance.

These patterns suggest that even when participants had access to many training exemplars, they selectively learned only from a subset of particularly valuable ones. The fact that, in both experiments, a DNN trained on the good set provides the best account of participants’ reaction times is, to us, both surprising and intriguing. It provides compelling data consistent with the runtime-learning hypothesis, and suggests that DNNs are capturing the role of the training set beyond mere performance.

Why would people use (primarily or exclusively) only a limited selection of training data? Assuming training and test instances are drawn from the same distribution, the theoretically optimal procedure (from a conventional ML perspective) is to use all data. When we consider conventional ML procedures, however, we see reason to believe this may not be sensible for people. Typically, ML systems are indeed trained on all available data but, importantly, they are trained such that they traverse the dataset *multiple times*. After a single traversal (or epoch), they are usually still far from their peak performance. Computers can store all examples in the training set in memory but, for people, the brain’s capacity to store every instance is probably much more limited. The brain is thus faced with a choice: either attempt to learn from the “true” data distribution (e.g., by learning from each training example only once) or store a few representative training instances and traverse this set multiple times. Unless the number of training examples presented is very small, the latter strategy is intuitively superior (given people’s attentional, memory, and other cognitive limitations), as it allows people to execute numerous training iterations over a small curated set of valuable training items stored in memory. Furthermore, the brain could keep the instances stored for use in training for a later task, an integral component of the runtime-learning hypothesis. Our results here suggest that the brain has adopted this latter strategy.

We therefore describe the hypothetical runtime learning process performed by the human participants in this experiment as follows. During training, each participant stores exemplar representations of each of the concepts (as in exemplar models). At each trial, the exemplar is either added to the collective representation, or used to modify one or more of the exemplars already in the representation, analogous to modern exemplar-based models of category understanding. This approach allows a good collective representation without storing every exemplar (which, as we just described, would be impractical). Simultaneously, the exemplars currently in the representation are being continuously used to train a discriminative neural network. As the representation becomes better and better, the training of the discriminative network will quickly become dominated by the valuable exemplars. With sufficient training—which may or may not have occurred in this particular experiment—generative models of the concepts may also be constructed and retained in long-term memory, to later be summoned when encountering a task involving them and used to produce exemplars, as in the first experiment.

### 3.5 Experiment 3

To support the runtime-learning hypothesis, we have relied primarily on two forms of evidence: correlations between DNN confidences and both participant reaction times and fitted drift rates, and DNN performance when trained on subsets of exemplars identified by analyzing participant reaction time. Concerning the former, although DNNs trained with the good training set provided the best account of participant reaction time data, it is not necessarily true that this actually tells us anything definitive about any purported subset used for internal training. For the latter, while we have demonstrated that the exemplars participants responded most quickly and accurately to form a good training set for training DNNs (relative to other equal-sized sets), it is not inevitable that humans will show this same property when trained on a comparable task. To address these concerns, we conducted an experiment in which we explicitly manipulated the training sets used by participants.

#### 3.5.1 Analysis

We scored each participant’s test data for accuracy. To determine whether the assigned training set had a significant effect on test-set accuracy, we conducted a t-test comparing the accuracies of participants trained on the good and bad training sets. Unlike in previous analyses, we did not apply a performance threshold for inclusion in the analysis, as this time performance was the key measure we were interested in. We found that participants trained on the good set scored significantly higher than participants trained on the bad set (mean 51.3% for the good set, 36% for the bad set, *p* < .01), indicating that participants (like DNNs) learn better from high-confidence exemplars.

We next calculated the Spearman rank correlations between DNN confidences and participants reaction times, as was done in the analyses of Experiments 1 and 2. Unlike the analyses of previous experiments, here we used the reaction times of participants with test-set accuracies of at least 50%—as opposed to the 75% threshold used previously—reflecting the brief training phase of this experiment which increased the difficulty of learning the classification task. This left 15 participants trained on the good set and 9 trained on the bad set. (We recognize that this is a small sample size, but the difficulty of this experimental condition makes it inherently difficult to obtain participants with good classification performance, as most participants do not do well. Fortunately, this portion of Experiment 3 is actually not the truly important part—what matters is the fact that participants trained on the good subset perform better than participants trained on the bad subset, a result derived from much more data.)

**Fig 8:**
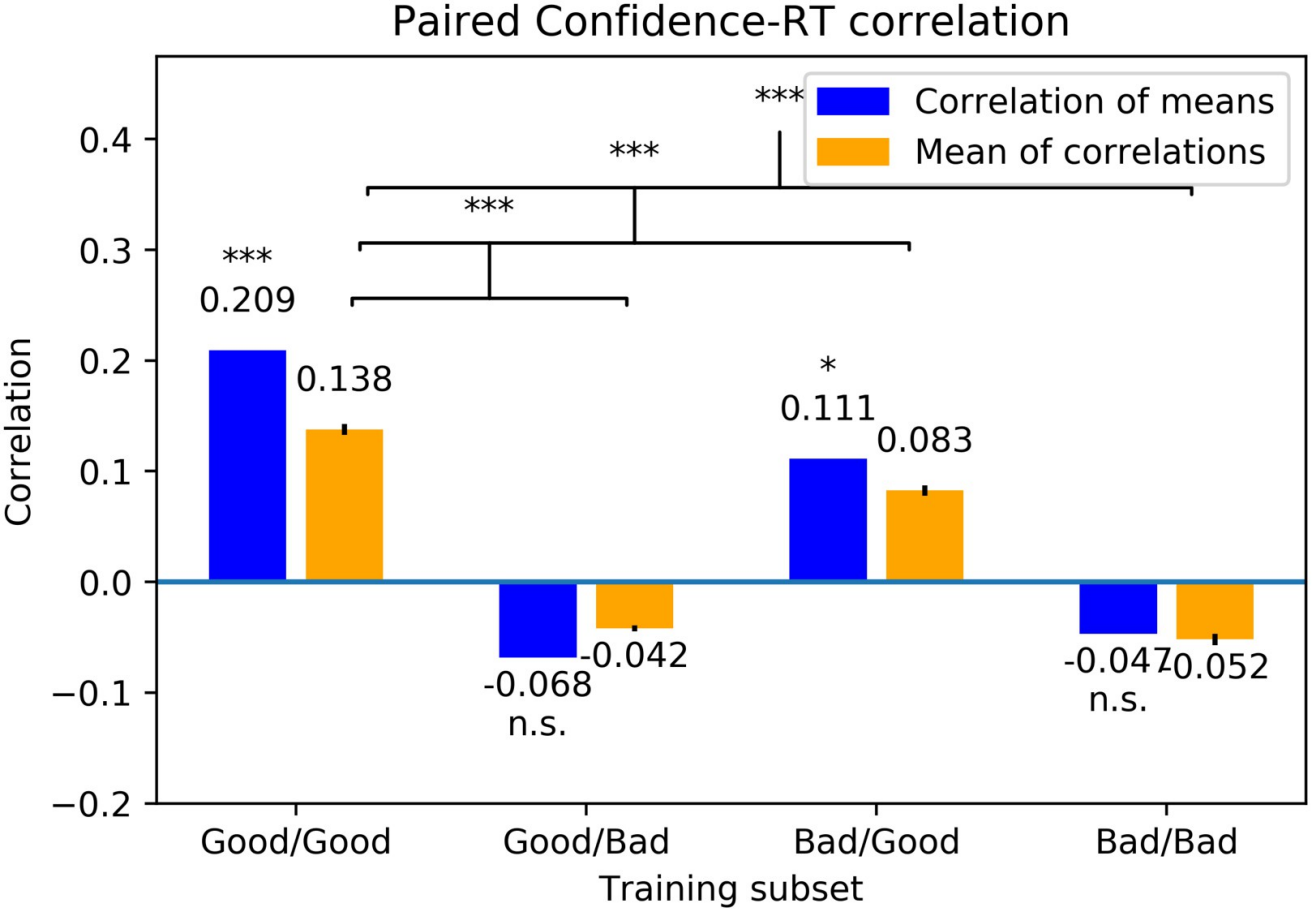
Correlation between human reaction time and DNN confidence for different training set-training set pairs. Spearman rank correlation coefficients between mean network confidences and participants’ mean normalized reaction times. The four pairs of bars correspond to the four possible combinations of DNN training (good versus bad training sets) and participant training (good versus bad training sets), with the first word in a label indicating the DNN training condition and the second word indicating the participant training condition. Averages were computed based on training one hundred randomly-initialized DNNs in each combination. Asterisks indicate the level at which the correlation was significantly different from zero; asterisks on brackets indicate the level at which pairs of sets of correlations are different from each other: ***: *p* < 0.001; **: *p* < 0.01; *: *p* < 0.05; n.s.: *p* > 0.05.

The results are shown in Fig 8. The four pairs of bars in the graph correspond to the four possible combinations of DNN training and participant training. For example, the first bar pair, labeled “Good/Good”, shows the Spearman’s correlation (averaged across one hundred runs of randomly initialized DNNs) between mean confidences of DNNs trained with the good training set (including data augmentation) and mean normalized reaction times of participants trained with the good training set, along with the mean of the individual correlations. For participants trained with the good set, DNNs trained with the good set provided better accounts of their reaction times than DNNs trained with the bad set. In contrast, for participants trained with the bad set, DNNs trained with good or bad sets both provided poor accounts of the reaction time data.

#### 3.5.2 Discussion

The results validate the analyses performed for the earlier experiments: not only do DNNs learn better from the exemplars participants respond to most quickly and accurately, participants themselves also learn better from those same exemplars. Although this is “external” learning, and not the “internal” learning that is central to the runtime-learning hypothesis, the results support the plausibility of the hypothesis that people store especially valuable exemplars for on-the-fly internal training.

Another interesting result is that we validated the use of reaction time correlations with neural network confidence as a method of uncovering the exemplars used in this internal training. The method correctly identified networks trained on the good dataset as providing the best account of the reaction times of participants trained on the good dataset. Just as importantly, these networks did not provide as good an account for the reaction times of participants trained on the bad dataset. The fact that even networks trained on the bad dataset did not provide an especially good account of participants trained on the bad dataset is perhaps not surprising, considering how poorly those networks generalize. If training on bad exemplars results in not only poor but also inconsistent performance, both by neural networks and humans, we would not expect to see correlations. Considering how difficult our participants evidently found the task when trained with the bad subset, they may have been unable to “converge” to similar “bad” solutions—after all, there are many more ways to *misunderstand* a task than to understand one, and with minimal experience on the poor exemplars, they may have focused on a variety of different “alternative” features. If they all (mis)understood the relevant concepts in different ways (choosing different features), then once aggregated we would not expect there to be much chance of significant correlations, since their judgments did not align in a meaningful way. It is also true that the bad correlations were already weaker in previous experiments, making them more susceptible to this issue. In any event, the results are consistent with the idea that the correlations found in earlier experiments may indeed reflect the effective training sets of the human participants.

## 4 General discussion

In summary, we have proposed the runtime-learning hypothesis which states that people handle idiosyncratic or unfamiliar tasks by drawing on stored instances of concepts relevant to the task to rapidly learn the appropriate function. Unless the task is predicted ahead of time, this learning takes place *after* the task is first presented. To make learning fast enough for this to be feasible, the hypothesis claims that only a few stored class instances are used, but these instances are especially valuable for training. We initially motivated the hypothesis by describing related ideas from the cognitive science and machine learning literatures. If people maintain a store of valuable examples of various (overlapping and hierarchical) categories, along with attribute labels and other knowledge, then even if a task has never been encountered before, a person can quickly organize a training set of valuable examples tailored to that task, possibly by using symbolic or graph neural network data structures and reasoning operations of the type that have been studied in cognitive science and AI for many years [4–8]. Using computer simulation, we showed that DNNs can learn effectively from small, curated training sets, and that valuable training items tend to lie toward the centers of data item clusters in an abstract feature space (S2 Appendix). Using behavioral experiments, we showed that people too can learn effectively from small, curated training sets. Perhaps the most compelling behavioral result is that people’s reaction times and fitted drift rates are best accounted for by the confidences of DNNs trained on small datasets of highly valuable items.

These results are exactly what the runtime learning hypothesis would predict, and at the same time the runtime learning hypothesis provides the best explanation for them that we are aware of. (A potentially contradictory result would have been the full dataset providing the best account, as this would have indicated that people may make their decisions based on extensive training on the task that may not be possible to perform rapidly. The random subset being as effective as the good subset in explaining the data would also be problematic, as it would indicate that people do not do the logical thing and use the best exemplars for rapid learning.) Thus, we believe it warrants further investigation. If the hypothesis is accepted, it suggests a number of topics for further research, in particular the precise nature of the internal computational learning algorithm and final decision process, both of which the hypothesis as it stands is mostly agnostic to. Hopefully, by carefully examining human perceptual phenomena, it will be possible to not only fine-tune the runtime learning hypothesis, but to in turn use it to even more accurately model human perception.

Note that we are not making any strong claims about the nature of the models that are trained at runtime. They may be neural networks resembling those we have used here, or may use a comparison process similar to that seen in models such as SUSTAIN. Our essential point is that humans appear to make their judgments based on a limited number of representative exemplars, which we explain as the result of having to train a model in a short period of time, presumably recently, in response to the task itself. If this were not the case, we would expect the results, at least for MNIST, to reflect the full subset, but in fact our data suggests humans use relatively limited subsets whether the concepts involved in the tasks are recently learned or have been highly familiar to the participants for years. The fact that this pattern was seen even with the Arabic digits, in which the concepts are assuredly familiar enough for our participants to use a model trained on many more exemplars, might be explained by saying that that the unfamiliar nature of the task itself (that is, the unique combination of digits that needed to be distinguished, along with other idiosyncratic circumstances) led to the “last minute” creation of a dedicated model, as stated by the runtime learning hypothesis. Beyond this, we are largely agnostic as to the precise form this “inner model” takes.

A natural question the reader may have concerns how the brain acquires the valuable exemplars that make training more effective. While this is not our primary concern here (although it may be a fruitful topic for future research), we would like to briefly discuss a few candidates. As mentioned previously, one of the major inspirations for this work was *data distillation* from the machine learning literature, and it is a distinct possibility that the brain employs a process resembling one data distillation algorithm or another to whittle a large corpus down to its essence. Another possibility is Generative Teaching Networks [15], an algorithm for learning generative models that produce exemplars that are especially valuable for learning. In fact, considering that the biases of GANs can naturally lead to “prototype”-like output (as demonstrated by mode collapse), it might even be that no special purpose-made algorithm is necessary to produce especially valuable exemplars.

The runtime-learning hypothesis also has implications for many other aspects of human cognition, including learning, memory, and attention. For brevity, we conclude by focusing on its implications for reasoning. Training with a small, curated training set is, of course, not usually as effective as training with *all* relevant training items. For the purposes of cognitive science, this might be a feature of the runtime-learning hypothesis, not a bug—after all, people’s task performances are often suboptimal [88], and people often tradeoff accuracy for speed [89]. Relatedly, several cognitive scientists have used sampling-based algorithms to perform approximate Bayesian inference in order to provide accounts of this suboptimality (e.g. [90–92]), and the runtime-learning hypothesis can also be viewed as implementing a sampling-based approach to approximate Bayesian inference. By sampling training exemplars from memory, one is implicitly sampling functions from an implicit distribution. Interestingly, the disparity between this sampling distribution and the true posterior may result in “biases and heuristics” similar to those observed in people [88]. For example, if stored exemplars are especially high-quality, as we have supposed throughout this paper, we would expect a learner to exhibit use of something similar to the representativeness heuristic [88]. In addition, it may be possible to account for the dynamic effects of expectation on perception [93–95] in a Bayesian manner by including instances of the classes in the training set in proportion to their prior probabilities.

This observation highlights a relationship between the runtime-learning hypothesis and a more general approach to studying human cognition known as the “resource rational” approach [89]. According to the resource-rational approach, people’s cognitive behaviors may be suboptimal when compared to the best performance achievable by an agent with unlimited internal (e.g., memory, attention) and external (e.g., time) resources, but these behaviors may be optimal when compared to the best performance achievable by an agent with limited resources. Researchers have used this approach to explain the apparent suboptimality of people’s decisions in some circumstances. For example, if one decision-making strategy is less accurate than another but is quicker and easier to implement, then a person may prefer this seemingly suboptimal strategy.

Although there is suggestive evidence that people complete tasks in a resource-rational manner [96–99], there are few mechanistic accounts of how this may occur. In our proposal, people can flexibly determine how to allot their cognitive resources in response to a task based on both the importance of the task and the time available before a response is required. For low-importance tasks, or tasks requiring a rapid response, people may opt to train a small network on only a few examples; conversely, for high-importance tasks and/or tasks which do not require a swift response, people may elect to train a more powerful network with more examples. Learning at runtime allows performance to be optimized not only for the task, but for the wider context in which a task is encountered.

## 5 Conclusions

Our runtime-learning hypothesis, along with the potential extensions described above, should be broadly understood as implying a spectrum upon which spontaneously-arising tasks may reside. At one extreme, there may already exist in the brain a network capable of handling precisely that task, meaning no runtime learning is necessary. At the other extreme, a task may be fully unfamiliar, with no previous experience with similar tasks, meaning the brain must rely entirely upon runtime learning from scratch. In between these two extremes lie cases in which the brain has some experience with similar tasks, but not enough to perform satisfactorily on this new task. Under these circumstances, techniques such as meta-learning can be used to accelerate the runtime learning process and improve the ultimate results. We speculate that most everyday but non-trivial cognitive tasks fit somewhere in this middle ground.

[100] recently pointed out that an important but underutilized path to producing general artificial intelligence is to create ML algorithms that learn how to produce algorithms for AI. This notion is present in the complementary learning systems approach in which a fast, rote learner teaches a slow, abstract learner. We propose that, in fact, algorithms that train other algorithms are essential to truly general intelligence, whether biological or artificial.

## Supporting information

S1 Appendix Machine learning literature. In this appendix, we describe the relevant machine learning literature in more detail.
(PDF)

S2 Appendix Additional computer simulations. We perform additional computer simulations.
(PDF)

S3 Appendix Modeling fitted drift rates with network confidences. We further analyze the correspondence between humans and models using a drift diffusion model.
(PDF)

S4 Appendix Analysis variations. We report variations of the analysis we performed in the main paper intended to reduce the probability that the results of our correlation analysis are due to artifacts unrelated to the training dataset.
(PDF)
