# Supplementary material for: Rapid runtime learning by curating small datasets of high-quality items obtained from memory
Source: PLoS Comput Biol. 2023 Oct 4;19(10):e1011445. doi: 10.1371/journal.pcbi.1011445 (PMC10578607; doi:10.1371/journal.pcbi.1011445)
Supplement: S1 Appendix — In this appendix, we describe the relevant machine learning literature in more detail. (PDF) [file pcbi.1011445.s001.pdf]

S1 Appendix: Machine Learning Literature

In this appendix, we describe the relevant machine learning literature in more detail.

S1.1 Dataset distillation

ML researchers often train their deep neural networks (DNNs) using tens of millions of data items, a time-consuming process that can take multiple days or weeks, not to mention the difficulty in acquiring so many examples. In many circumstances, people cannot take the time to train on so many examples if they hope to produce a network quickly enough to respond to a task in a timely manner. Some researchers have noted that data items in a training set tend to be at least partially redundant. Thus, it may be possible to efficiently train learning systems using a small set of especially valuable items (e.g., [1–6]). This would be worthwhile so long as the systems achieve good performance, even if this performance is suboptimal (relative to training using *all* data items); after all, performing a task at an above-chance level is better than not performing it at all.

As described in the main text, the ML literature includes methods both to identify a small set of valuable training items from a large set (sometimes referred to as "core-set selection"; see [4, 5]) and methods to synthesize a small set of valuable training items. For convenience, we refer to both types of methods as "dataset distillation" [6]. Methods to identify a small set of valuable training items often attempt to discover clusters of items in the original large set, and then select items near cluster centers for inclusion in the small set. Based on this intuition, Sener and Savarese [4] developed an "active learning" (see main text) approach to identify valuable training items for training convolutional neural networks. Tsang et al. [5] used a related idea to identify valuable training items for kernel-based methods (e.g., support vector machines) which typically cannot be efficiently trained with very large datasets.

A common approach to synthesizing small sets of training items is to treat the set of training examples as parameters to optimize during meta-training (i.e., the examples are adjusted to

maximize the performance of a learning system trained on them). Kusner et al. [2], for instance, proposed "stochastic neighbor compression", an algorithm that synthesizes a small set of training items from a large set. When the small set is used with a probabilistic nearest neighbor classifier, it minimizes the classifier's error on the large set. The authors report that their technique speeds up classification (by up to several orders of magnitude) and yields robustness to label noise (even when the small set is only 2% of the size as the large set). Wang et al. [6] used a similar idea, but with DNNs. They synthesized ten images (one per class) from the 60,000 MNIST training images, and trained a DNN using these ten images to achieve performance nearly as good as the performance of a DNN trained with the full dataset.

S1.2 Meta-learning and few shot-learning

The form of meta-learning most relevant to our runtime-learning hypothesis is known as *few-shot learning*. Here, the goal is to use labeled training data on multiple tasks to learn to learn new tasks with only a few exemplars. For instance, "model-agnostic meta-learning" (MAML [7]) is a method in which a model is trained with a loss function that depends on the model's performance on many tasks. The hope is that the method will converge on a set of initial parameter values that can be quickly "fine-tuned" to enable the model to perform well on any of them, or on a novel (but similar) task, using only a few examples.

Another relevant approach is that of Vinyals et al. [8], who augmented a neural network with memory to form "matching networks". A matching network learns to become a nearest neighbor classifier with a task-independent embedding function obtained through training on multiple tasks. When given a new task, meaning a novel set of labeled training items and a set of unlabeled test items, a network embeds the test items and the (small set of) training items and uses its similarity function (cosine distance in the original paper) to predict the classes of test items by comparing the embedded training and test items. Santoro et al. [9] also explored few-shot learning based on memory, making use of neural Turing machines (NTMs [10]). NTMs include a network that learns when and what to read and write to a memory. Santoro et al. [9] demonstrated that they can learn

representations and read/write rules to enhance learning on novel tasks. Many other meta-learning algorithms make use of memory [11].

A different, but also relevant, approach to few-shot learning is that of Antoniou et al. [12], who used generative adversarial networks (GANs; [13]) to augment the (small amount of) training data provided for a novel task. Labeled data was used to train a GAN so that when it was conditioned on an instance of a class, it could produce additional samples of the class. To learn a novel class from one or a few training instances, the trained GAN is conditioned on the new instance(s) and the resulting samples are used to augment the training data for the novel class. Essentially, the GAN learns identity-preserving transformations that can be used to effectively augment a small training set. Wang et al. [14] used a similar approach, but instead of training adversarially, a generator was trained to produce samples to improve another network's classification performance when those samples were added to the network's training set. In yet another variation, Schwartz et al. [15] used a modified autoencoder to produce the transformations. For a more comprehensive review of meta-learning, we recommend Vanschoren [16] and Wang [11].

Some readers may wonder if runtime learning is truly necessary considering recent advances in few-shot learning, such as those exhibited by language models such as GPT-3 [17]. However, black-box few-shot learning approaches such as GPT-3 still often make errors that can seem inexplicable or laughable—or even worse, offensive and potentially harmful—to humans. Although these issues may be solved by continued work in the field, we believe is prudent to continue exploring other possibilities as well. None of this is intended to diminish the impressive feats and brilliant engineering underlying GPT-3, and we believe that it and similar few-shot algorithms may have a great deal to tell us about human cognition.

Besides, these advances still assume that external exemplars are explicitly provided when new tasks are to be performed. For instance, one demonstration of GPT-3's few-shot learning capability involved a request for English-French translation, accompanied by a few English/French pairs as examples. Rather than competing with these methods, runtime learning (being inner-model-agnostic) would propose that these algorithms can also be put to use with *internal* exemplars and

tasks. Runtime learning would suggest that upon being asked to translate a novel word, the brain could summon a few established English/French pairs and present them to a GPT-3-like model, which can then translate the requested word with their help. Again, since run-time learning is agnostic to the composition of the inner model, the fact that GPT-3 does this without gradient updates is irrelevant. We would be interested to see the behavior of a GPT-3-like model specifically trained to take advantage few-shot exemplars.

We have already begun work examining the use of various few-shot algorithms in runtime learning. It is even possible that the added structure that runtime learning provides (such as through the use of a symbolic reasoning process) could help alleviate the problems seen in these large few-shot learners, such as the bizarre non-sequiturs and apparent blindness to relevant information observed in GPT-3. As a matter of fact, even now, runtime learning may not even be competing with black-box few-shot learners such as GPT-3. After all, it is not entirely clear exactly how deep neural networks, let alone especially large and complex ones such as GPT-3, even work at a detailed level, beyond the basic building blocks such as transformers. This is all very well and good from an engineering perspective, which focuses on practical results, but as scientists we would like to know how the mind works in more detail. It is possible that GPT-3 employs a process not dissimilar to runtime learning to accomplish its feats. This is especially plausible considering the amount of data stored in the network's parameters (over 800 gigabytes) and that the transformer mechanism it is based on is inherently capable of selective attention.

S1.3 Generative models and GANs:

In contrast to discriminative models, which learn the distribution of classes given observations, generative models learn the joint distribution of classes and observations. When combined with a prior distribution over classes, sampling from a generative model conditioned on a class produces samples that are likely to belong to the class. In addition to this sampling ability, generative models have the advantage of learning the "hidden causes" of the observations, making them well-suited for unsupervised learning.

Currently, the most popular form of generative model is the "generative adversarial network" (GAN; [13]), which learns to generate samples of a class using two networks: one of which (the generator) is given a random noise vector as input and produces an output vector, and another (the discriminator) which attempts to determine whether its input is a genuine example of the class from a training set or something produced by the generator. The two networks are trained adversarially, with the generator trying to fool the discriminator and the discriminator trying not to be fooled. After training, the samples produced by the generator network can appear to people to be valid examples of the class (although the best way to evaluate generative models remains a significant issue; [18]).

We believe that generative models are important to generalizing runtime learning beyond relatively simple tasks such as the ones used in this paper, and indeed in machine learning in general, to naturalistic stimuli. For example, image-to-image generative models such as CycleGANs [19] could be used to produce exemplars of objects in varying contexts. To produce exemplars of "dogs on the beach", a generative model of dogs could produce exemplars of dogs on a "blank" background, then another generative model can be used to place that dog on a beach. Depending on the compositionality of a concept and the generative models, exemplars of concepts could be produced without either stored exemplars of that particular concept or a dedicated generative model of that concept. Also relevant to this topic would be generative methods of zero-shot learning, which we discuss in the next section.

S1.4 Zero-shot learning

Zero-shot learning is even more extreme than few-shot learning: it is the ability to learn concepts without any exemplars at all. It usually involves a semantic space into which concepts can be placed. Exactly how this is done varies widely in the ML literature. One approach, seen in the models of, among others, Palatucci et al. [20] and Socher et al. [21], is to learn a mapping from stimuli to codes. A new class can be learned without additional training data by providing a semantic description of the new class. A novel stimulus is assigned to this new class when its

semantic code matches the class's semantic description. Put another way, labeled classes are used to teach a system to produce a mid-level description of a stimulus (for example, a network might learn to describe a dog as "furry", "quadrupedal", but not "feathered" or "bipedal", etc.). The system can then be provided with a description of a novel class making use of the same attributes (such as "feathered", "bipedal", and not "quadrupedal" or "furry" for the concept "bird"), and it can determine whether stimuli belong to that class by comparing their encodings to the description (an image of a bird is identified as a bird if the encoding of the image is a sufficiently close match to the provided description).

In terms of relevance to our own work, special mention should be made of "feature generating networks" [22], which learn to produce synthetic examples of feature embeddings of class members conditioned on an attribute-based description of the class. These examples are used to improve the performance of a discriminator trained on them. The generative network can then be used to produce samples of unseen classes by conditioning its output on an attribute-based description of the novel class. A discriminator network can then be trained to recognize this novel class using the synthetic examples. For a more comprehensive review of zero-shot learning, we recommend Xian et al. [23].

References

[1] Birodkar V, Mobahi H, Bengio S. Semantic redundancies in image-classification datasets: The 10% you don't need. arXiv preprint arXiv:190111409 2019;.

[2] Kusner M, Tyree S, Weinberger K, Agrawal K. Stochastic neighbor compression. In: Proceedings of the 31st International Conference on Machine Learning; 2014. p. 622–630.

[3] Lapedriza A, Pirsiavash H, Bylinskii Z, Torralba A. Are all training examples equally valuable? arXiv preprint arXiv:13116510 2013;.

[4] Sener O, Savarese S. Active learning for convolutional neural networks: A core-set approach. Proceedings of the International Conference on Learning Representations 2018;.

[5] Tsang I, Kwok J, Cheung PM. Core vector machines: Fast SVM training on very large data sets. Journal of Machine Learning Research 2005;6:363–392.

[6] Wang T, Zhu JY, Torralba A, Efros AA. Dataset distillation. arXiv preprint arXiv:181110959v3 2020;.

[7] Finn C, Abbeel P, Levine S. Model-agnostic meta-learning for fast adaptation of deep networks. arXiv preprint arXiv:170303400 2017;.

[8] Vinyals O, Blundell C, Lillicrap T, Kavukcuoglu K, Wierstra D. Matching Networks for One Shot Learning. In: Lee D, Sugiyama M, Luxburg U, Guyon I, Garnett R, editors. Advances in Neural Information Processing Systems, vol. 29 Curran Associates, Inc.; 2016. p. 3630–3638.

[9] Santoro A, Bartunov S, Botvinick M, Wierstra D, Lillicrap T. Meta-learning with memory-augmented neural networks. In: Proceedings of the 33rd International conference on machine learning; 2016. p. 1842–1850.

[10] Graves A, Wayne G, Danihelka I. Neural Turing machines. arXiv preprint arXiv:14105401 2014;.

[11] Wang JX. Meta-learning in natural and artificial intelligence. arXiv preprint arXiv:201113464 2020;.

[12] Antoniou A, Storkey A, Edwards H. Data augmentation generative adversarial networks. arXiv preprint arXiv:171104340 2017;.

[13] Goodfellow I, Pouget-Abadie J, Mirza M, Xu B, Warde-Farley D, Ozair S, et al. Generative Adversarial Nets. In: Ghahramani Z, Welling M, Cortes C, Lawrence N, Weinberger KQ, editors. Advances in Neural Information Processing Systems, vol. 27 Curran Associates, Inc.; 2014. p. 2672–2680.

[14] Wang YX, Girshick R, Hebert M, Hariharan B. Low-shot learning from imaginary data. In: Proceedings of the 2018 IEEE/CVF conference on computer vision and pattern recognition; 2018. p. 7278–7286.

[15] Schwartz E, Karlinsky L, Shtok J, Harary S, Marder M, Kumar A, et al. Delta-encoder: an effective sample synthesis method for few-shot object recognition. In: Bengio S, Wallach H, Larochelle H, Grauman K, Cesa-Bianchi N, Garnett R, editors. Advances in Neural Information Processing Systems, vol. 31 Curran Associates, Inc.; 2018. p. 2845–2855.

[16] Vanschoren J. Meta-learning: A survey. arXiv preprint arXiv:181003548 2018;.

[17] Brown T, Mann B, Ryder N, Subbiah M, Kaplan JD, Dhariwal P, et al. Language models are few-shot learners. Advances in neural information processing systems 2020;33:1877–1901.

[18] Theis L, Oord Avd, Bethge M. A note on the evaluation of generative models. arXiv preprint arXiv:151101844 2015;.

[19] Zhu JY, Park T, Isola P, Efros AA. Unpaired image-to-image translation using cycle-consistent adversarial networks. In: Proceedings of the IEEE international conference on computer vision; 2017. p. 2223–2232.

[20] Palatucci M, Pomerleau D, Hinton GE, Mitchell TM. Zero-shot Learning with Semantic Output Codes. In: Bengio Y, Schuurmans D, Lafferty J, Williams C, Culotta A, editors. Advances in Neural Information Processing Systems, vol. 22 Curran Associates, Inc.; 2009. p. 1410–1418.

[21] Socher R, Ganjoo M, Manning CD, Ng A. Zero-Shot Learning Through Cross-Modal Transfer. In: Burges CJC, Bottou L, Welling M, Ghahramani Z, Weinberger KQ, editors. Advances in Neural Information Processing Systems 26 Curran Associates, Inc.; 2013.p. 935–943.

[22] Xian Y, Lorenz T, Schiele B, Akata Z. Feature generating networks for zero-shot learning. In:

Proceedings of the 2018 IEEE/CVF conference on computer vision and pattern recognition; 2018. p. 5542–5551.

[23] Xian Y, Lampert CH, Schiele B, Akata Z. Zero-shot learning—A comprehensive evaluation of the good, the bad and the ugly. IEEE Transactions on Pattern Analysis and Machine Intelligence 2018;41(9):2251–2265.
