# Supplementary material for: Rapid runtime learning by curating small datasets of high-quality items obtained from memory
Source: PLoS Comput Biol. 2023 Oct 4;19(10):e1011445. doi: 10.1371/journal.pcbi.1011445 (PMC10578607; doi:10.1371/journal.pcbi.1011445)
Supplement: S2 Appendix — We perform additional computer simulations. (PDF) [file pcbi.1011445.s002.pdf]

S2 Appendix: Additional Computer Simulations

S2.1 Datasets based on classifier responses

To extend the results reported above, we also created training sets in which data items were selected based on ResNet-50's performance. First, ResNet-50 was trained on the entire CIFAR-10 training set. Then the CIFAR-10 test set was randomly split into an evaluation set (with 400 items per category) and a new test set $\mathcal{D}_T$ (with the remaining 600 items per category). ResNet-50's performance on each of the items in the evaluation set was calculated (using the cross-entropy loss function). For reasons described below, we also evaluated ResNet-50's performance on each item in $\mathcal{D}_T$ in order to create two new test sets, denoted $\mathcal{D}_{EASY}$ and $\mathcal{D}_{HARD}$, each containing the 50 test items from each category on which ResNet-50 performed best and worst, respectively.

Items from the evaluation set on which ResNet-50 performed well were placed in a high-quality training set $\mathcal{D}_{HQ}$, whereas items in which it performed poorly were placed in a low-quality training set $\mathcal{D}_{LQ}$. A random training set $\mathcal{D}_R$ was also created by randomly selecting items from the CIFAR-10 training set. This procedure was repeated multiple times to create training sets of different sizes, ranging from 25 to 200 items per category. For each set size, ResNet-50 was trained with the corresponding training sets, and its accuracies were measured on test set $\mathcal{D}_T$.

The results are shown in Fig A. The horizontal axis of this graph shows the number of training items per category, and the vertical axis shows the test set accuracy following training. The blue, green, and orange lines correspond to training on high-quality, low-quality, and random items, respectively. As expected, test set accuracies increase as the sizes of the training sets increase. Critically for our purposes, training with high-quality items leads to better performance than training with random items when training sets are very small (e.g., 25-100 items per category), and leads to equal performance as random training with moderately small training sets (e.g., 125-200 items per category).

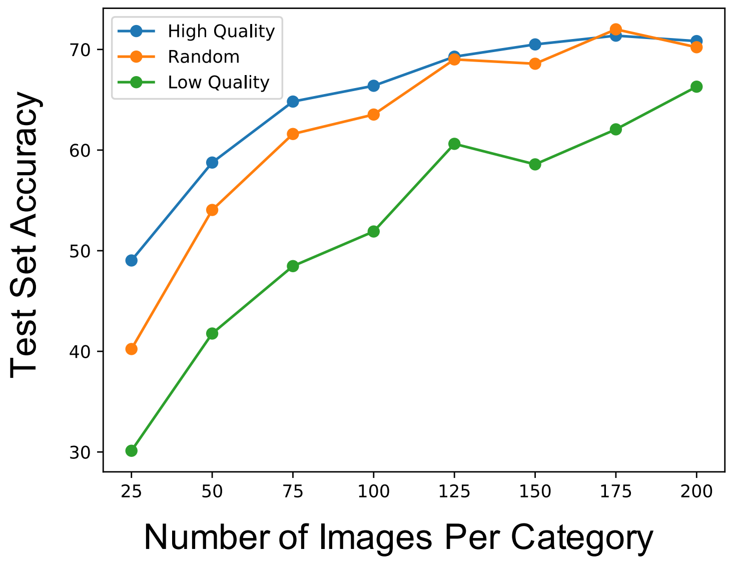

Fig A: Horizontal axis shows the number of items per category in a training set. Vertical axis shows the accuracy (percent correct) on test set $\mathcal{D}_T$ after ResNet-50 was trained on $\mathcal{D}_{HQ}$, $\mathcal{D}_{LQ}$, or $\mathcal{D}_R$.

S2.2 Origin of high performance with valuable exemplars

The results reported here are consistent with those reported above. As discussed above, these results may be considered surprising. Items in test set $\mathcal{D}_T$ were selected at random, and thus this set presumably contains a wide variety of CIFAR-10 items, including items that can be characterized as “easy”, “moderate”, and “hard”. When trained with random dataset $\mathcal{D}_R$, ResNet-50 experienced all types of items. However, when trained with high-quality dataset $\mathcal{D}_{HQ}$, it never experienced “hard” items. Despite this lack of experience, its test performance is better when trained with very small high-quality sets than with very small random sets.

To better understand this apparent paradox, we evaluated ResNet-50’s performance on biased test sets $\mathcal{D}_{EASY}$ and $\mathcal{D}_{HARD}$ when trained with training sets of 25, 75, or 150 items per category. The results are shown in Fig B. On test set $\mathcal{D}_{EASY}$, training with $\mathcal{D}_{HQ}$ outperforms training with $\mathcal{D}_R$, as expected. In contrast, on test set $\mathcal{D}_{HARD}$, training with $\mathcal{D}_R$ tends to be superior, again as expected.

Given these results, why does training with high-quality items often lead to better test perfor-

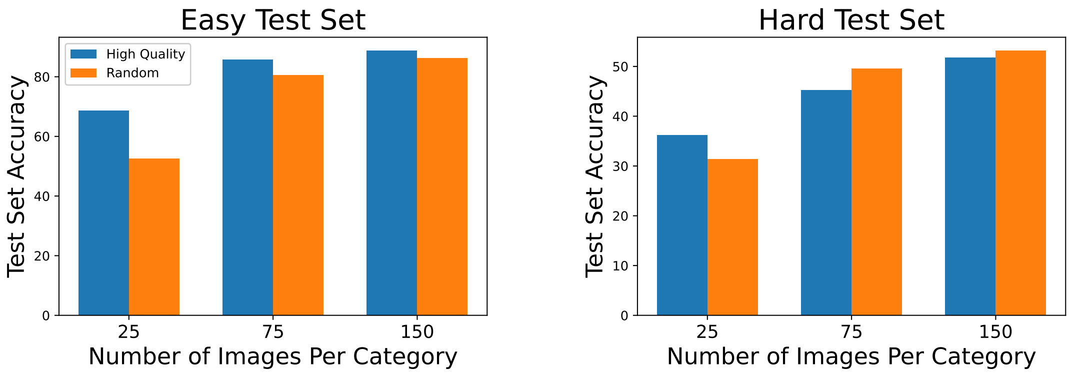

Fig B: ResNet-50 accuracies (percent corrects) after training on $\mathcal{D}_{HQ}$ (blue bars) or $\mathcal{D}_R$ (orange bars) when training sets contained 25, 75, or 150 data items per category. Accuracies were evaluated on test sets $\mathcal{D}_{EASY}$ (left graph) and $\mathcal{D}_{HARD}$ (right graph).

mance than training with random items (Fig A)? We conjecture that, because of the categorical structure of CIFAR-10, data items are clustered in some abstract feature space. When learning from a limited number of training examples, the most important task may be to learn these cluster centers (consider the extreme situation of constructing a 1-nearest-neighbor classifier with only one exemplar per class; intuitively, we should put each exemplar at the center of the class cluster). Although randomly-selected exemplars potentially reflect the true distribution more accurately, they may make it more difficult to identify the cluster centers. On the other hand, high-quality training items are presumably near cluster centers, and thus help learning systems quickly learn cluster locations. This crucial property is especially important when training sets are very small; once sets reach a certain size, the ability to also cover edge cases farther from the cluster center becomes more important than just putting even more weight on the center.

To evaluate the relative locations of CIFAR-10 images in a suitable abstract feature space, we first trained ResNet-50 on the entire CIFAR-10 training set (50,000 training items), and then visualized the hidden or latent representations of different subsets of the CIFAR-10 test images. For each test image, we formed a vector of the activations of units in the representational layer immediately before the final output layer. Using t-SNE, a nonlinear dimensionality-reduction technique used for visualizing high-dimensional data [1], we mapped these vectors to a two-dimensional space.

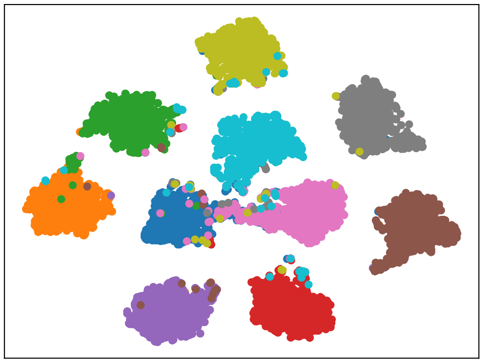

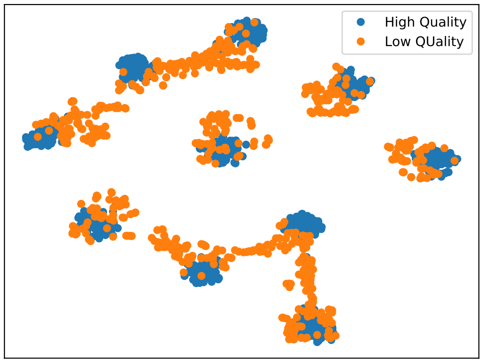

Fig C: Two-dimensional visualizations of test images based on ResNet-50 representations following training on all CIFAR-10 training images. (Left) Visualizations of evaluation images. (Right) Visualizations of images in $\mathcal{D}_{HQ}$ (blue) and $\mathcal{D}_{LQ}$ (orange).

The left graph of Fig C shows dots representing images from the evaluation set (400 images per category) in this space, where a dot's color is based on the corresponding image's category. Clearly, images fall into clusters, with one cluster for each category. The right graph is similar, except it visualizes the images in high-quality dataset $\mathcal{D}_{HQ}$ (blue dots) and low-quality dataset $\mathcal{D}_{LQ}$ (orange dots), each with 100 images per category. As expected, high-quality images tend to congregate near cluster centers.

For Fig D, we trained ResNet-50 on either high-quality dataset $\mathcal{D}_{HQ}$ or random dataset $\mathcal{D}_R$, each with 75 images per category. The left and right graphs in the top row visualize the images in $\mathcal{D}_{EASY}$ and $\mathcal{D}_{HARD}$, respectively, following training on $\mathcal{D}_{HQ}$. The graphs in the bottom row show the analogous information following training on $\mathcal{D}_R$. Images in $\mathcal{D}_{EASY}$ are more clearly clustered after training with $\mathcal{D}_{HQ}$ than after training with $\mathcal{D}_R$. The opposite pattern holds with images in $\mathcal{D}_{HARD}$ where clustering seems more evident following $\mathcal{D}_R$ training than following $\mathcal{D}_{HQ}$ training. Overall, these results are consistent with those in Fig B.

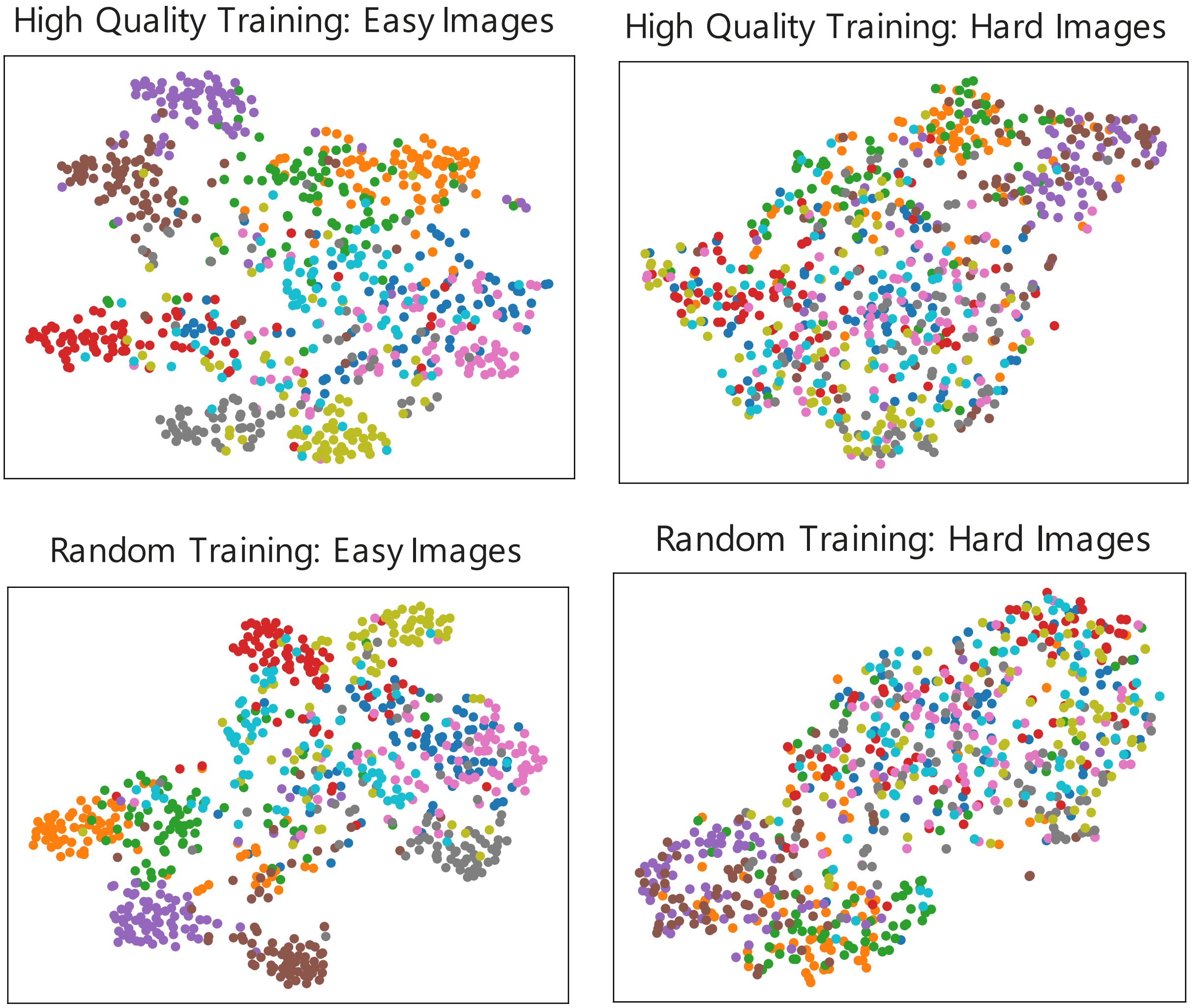

Fig D: Two-dimensional visualizations of images in $\mathcal{D}_{EASY}$ (left column) or $\mathcal{D}_{HARD}$ (right column) based on ResNet-50 representations following training on high-quality dataset $\mathcal{D}_{HQ}$ (top row) or random dataset $\mathcal{D}_{R}$ (bottom row).

References

[1] van der Maaten L, Hinton G. Visualizing data using t-SNE. Journal of Machine Learning Research 2008;9:2579–2605.
