# Supplementary material for: Rapid runtime learning by curating small datasets of high-quality items obtained from memory
Source: PLoS Comput Biol. 2023 Oct 4;19(10):e1011445. doi: 10.1371/journal.pcbi.1011445 (PMC10578607; doi:10.1371/journal.pcbi.1011445)
Supplement: S3 Appendix — We further analyze the correspondence between humans and models using a drift diffusion model. (PDF) [file pcbi.1011445.s003.pdf]

S3 Appendix: Modeling fitted drift rates with network confidences

Modeling fitted drift rates with network confidences: The assumption we made in the main body of the paper, namely that only drift rate changes from exemplar to exemplar, seems like a safe one, but we can eliminate it and more directly estimate the drift for individual exemplars by fitting an appropriate model to participant reaction time data. In consideration of the relatively small number of data points we have for each individual exemplar, we use a simple model based on the shifted Wald distribution. The Wald distribution, also (somewhat misleadingly) called the inverse-Gaussian distribution, describes the distribution of times at which a stochastic process with positive drift γ crosses some defined threshold α. The shifted Wald distribution simply adds an offset θ to the start of the process. The probability density function of the shifted Wald distribution is:

$$PDF(x|\alpha,\theta,\gamma) = \frac{\alpha}{\sqrt{2\pi(x-\theta)^3}} \cdot \exp - \frac{[\alpha-\gamma(x-\theta)]^2}{2(x-\theta)} \tag{1}$$

for $x > \theta$, where x denotes a reaction time.

Anders et al. [1] recommend the shifted Wald distribution as a simple, interpretable cognitive process model of response times that can be used in situations with relatively few data points. While it most directly applies to decisions with only a single possible response, such as go/no-go tasks, they also note that it can be applied to more complicated tasks to produce a relatively abstract aggregate description of the process producing the entire reaction time distribution. The entropy of neural network responses is a similarly aggregate measure of drift, so this is useful even if, as Matzke and Wagenmakers [2] found, the fitted shifted Wald drift only imperfectly corresponds to the drifts of the *individual* components for each possible decision in a more complicated model. Thus, we can abstractly interpret our task as having only a single outcome that represents *any* decision being made, without any other change in our earlier logic concerning the interpretation of the parameters. Therefore, for each exemplar, we performed maximum likelihood estimation using

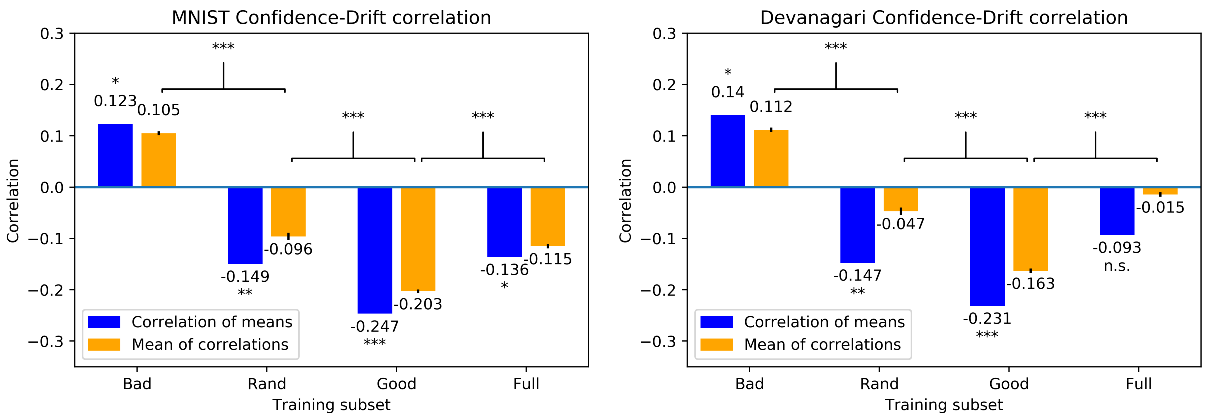

Fig A: Spearman rank correlation coefficients between the mean of networks' confidences and fitted drift values for MNIST (left) and Devanagari (right), based on training one hundred randomly-initialized networks on each training set. The left, blue bar is the correlation between the mean network confidence for each exemplar and mean human RT for the same exemplar; the right, orange bar is the mean of the correlations between each network's confidence and mean human RT, with error bars representing standard errors of the means. Asterisks above bars indicate the level at which the correlation was significantly different from zero; asterisks on brackets indicate the level at which pairs of sets of correlations are different from each other: $***$: $p < 0.001$; $**$: $p < 0.01$; $*$: $p < 0.05$; n.s.: $p > 0.05$.

the Broyden-Fletcher-Goldfarb-Shanno optimization algorithm to fit a shifted Wald distribution to the human reaction times (normalized as before) induced by the exemplar, where the main parameter of interest is the drift rate γ for each exemplar. We then found the Spearman's rank correlation coefficient between the drift for an exemplar and the mean normalized confidence, as well as the individual confidences, induced by the same exemplar in one hundred neural networks, set up as before and trained on each of the subsets, for both MNIST and Devanagari. Here, we expect a good fit to produce a *negative* correlation, as low entropy should correspond to a high drift rate.

As seen in Fig A (right graph), for both MNIST and Devanagari, we found a moderate but highly significant negative correlation for networks trained on the good set, lesser (and less significant) negative correlations for random sets and the full dataset, and a positive correlation for networks trained on the bad set. The correlations produced by the good set were also significantly different from the others. Thus, based on both the direction, magnitude, and significance of the correlations, networks trained on the good training set again provide the best account of partici-

pants' reaction times in our experiment. (We did not fit shifted Wald distributions and correlate the resulting drifts for Experiment 3, as we lacked enough data points per exemplar for a meaningful fit.)

References

[1] Anders R, Alario F, Van Maanen L, et al. The shifted Wald distribution for response time data analysis. Psychological Methods 2016;21(3):309–327.

[2] Matzke D, Wagenmakers EJ. Psychological interpretation of the ex-Gaussian and shifted Wald parameters: A diffusion model analysis. Psychonomic Bulletin & Review 2009;16(5):798–817.
