# Supplementary material for: Rapid runtime learning by curating small datasets of high-quality items obtained from memory
Source: PLoS Comput Biol. 2023 Oct 4;19(10):e1011445. doi: 10.1371/journal.pcbi.1011445 (PMC10578607; doi:10.1371/journal.pcbi.1011445)
Supplement: S4 Appendix — We report variations of the analysis we performed in the main paper intended to reduce the probability that the results of our correlation analysis are due to artifacts unrelated to the training dataset. (PDF) [file pcbi.1011445.s004.pdf]

S4 Appendix: Analysis Variations

In this appendix, we describe variations of the analysis we performed in the main paper intended to reduce the probability that the results of our correlation analysis are due to artifacts unrelated to the training dataset.

S4.1 Performance titration

Our results could conceivably be due to some sort of overfitting phenomenon, such that the models trained on the full dataset are less effective at describing human reaction time data solely due to their more extensive training. To put it another way, there may be a particular level of performance that enables a model to effectively explain human reaction time, while models that perform better or worse do not do as well, regardless of what training data was used to achieve that level. In this scenario, models trained on the good dataset succeed because they happen to hit this level of performance, whereas the other datasets result in performance that is too low or high. Our results would therefore be unrelated to the actual contents of the various training subsets.

To address this, we determined the amount of training required to bring models trained on random datasets with extra exemplars only to a level of performance similar to the good dataset. We created random training subsets with eight or six exemplars of each character class for MNIST and Devanagari respectively (rather than five, as with the other subsets), then titrated their training such that the models trained on them performed similarly to models trained on the good subset (still 64 epochs for MNIST, just 32 for Devanagari). For both MNIST digits and Devanagari characters, these titrated models did not explain the human data as well as the models trained on the good dataset.

The fact that two models can have similar accuracy but wildly different abilities to explain human data suggests that the differences in correlation we found are actually due to the differences in training set and, by extension, the features learned by the model.

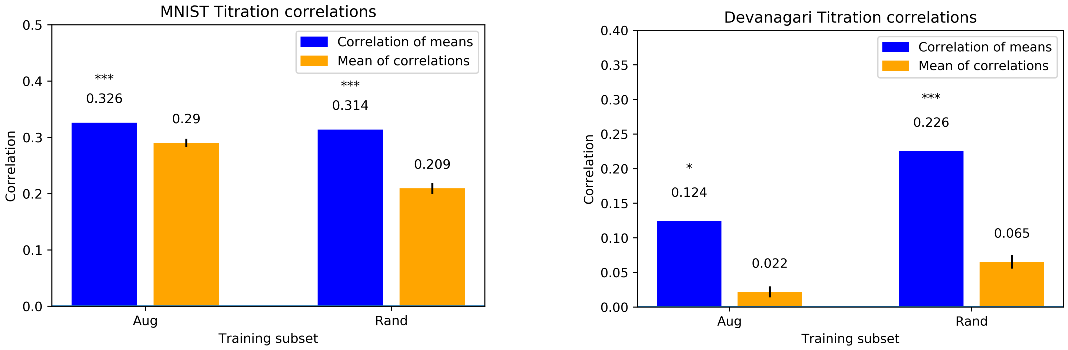

Fig A: The correlations produced with titrated models as described in the text, using 100 DNNs trained on each subset. "Aug" refers to the augmented dataset, consisting of exemplars that were at least rotated, and possibly also translated, for a total of 5760 exemplars. "Rand" refers to the the random dataset titrated to perform similarly to the corresponding good dataset. Asterisks above bars indicate the level at which the correlation was significantly different from zero: $***$: $p < 0.001$; $**$: $p < 0.01$; $*$: $p < 0.05$; n.s.: $p > 0.05$.

S4.2 Exemplar exposure

It is also possible that our results are due to the models memorizing their training sets, and the model trained on the full dataset being equally familiar (and therefore equally confident) with all the exemplars. Our analysis does not particularly support this interpretation; if this effect is present one would expect the full dataset to produce models with no correlation with the human data, but the models trained on the full dataset exhibited a significant correlation—just a lesser one than the good-trained models.

However, to be sure, we tried training the models exclusively on the results of data augmentation; that is, they were trained on rotated and translated exemplars, but not the unmodified originals, which therefore remained unfamiliar. More specifically, they were trained on exemplars that were at least rotated, and possibly also translated, for a total of 320 exemplars $\times$ 9 translations $\times$ 2 rotations (excluding the original orientation) for a total of 5760 exemplars. If our results are due to memorization, we would expect models trained on the augmentations of the full set to perform as well as the models trained on the good dataset. However, as seen in Fig A, we found that models trained solely on the augmentations (but still tested for their confidence on the originals) provided similar accounts of human reaction times as the corresponding datasets that included the originals,

so the original model trained the good dataset still produces the best correlations. This suggests that our results are probably not due to any memorization effect.
